# Adaptation and Validation of a Test for the Evaluation of Tactical Knowledge in Soccer: Test de Conocimiento Táctico Ofensivo en Fútbol for the Brazilian Context (TCTOF-BRA)

**DOI:** 10.3389/fpsyg.2022.849255

**Published:** 2022-07-14

**Authors:** Leandro Rechenchosky, Vanessa Menezes Menegassi, Matheus de Oliveira Jaime, Paulo Henrique Borges, Jaime Serra-Olivares, Wilson Rinaldi

**Affiliations:** ^1^Group of Studies and Researches Applied in Soccer (GEPAFUT), Department of Physical Education, State University of Maringá, Maringá, Brazil; ^2^Department of Physical Education, Federal University of Santa Catarina, Florianópolis, Brazil; ^3^Pedagogy in Physical Education, Faculty of Education, Catholic University of Temuco, Temuco, Chile

**Keywords:** validity, football, tactics, decision making, psychometrics, Brazil

## Abstract

**Background:**

Studies and tests to assess the tactical domain of young soccer players are recent, and few instruments meet the majority of quality criteria.

**Objective:**

To adapt and validate the *Test de Conocimiento Táctico Ofensivo en Fútbol* (TCTOF) for the Brazilian context (TCTOF-BRA).

**Methods:**

The article consists of two studies. Study 1 (*n* = 111) included the translation, theoretical/semantic analysis, back translation, cross-cultural equivalence, and content and face validity (pre-test). In study 2 (*n* = 768), a theoretical and empirical item analysis was carried out, followed by construct validity [exploratory factor analysis (EFA), confirmatory factor analysis (CFA), and the known-groups method] and reliability (internal consistency and repeatability).

**Results:**

In the cross-cultural evaluation, the Coefficient of content validity total (CCV_t_) of the instrument was 0.96 and in the content validity, the CCV_t_ of the instrument was 0.87. The face validity was confirmed (>95%). After theoretical and empirical analysis, 15 questions were included in the *Teste de Conhecimento Tático Ofensivo no Futebol* (TCTOF-BRA). The EFA showed a model with adequate fit (KMO = 0.69; *Bartlett p* < 0.001), with a factor structure considered very good, composed of four factors (decision making, operational tactical principles, collective tactical-technical elements, and rules). The CFA by the Asymptotically Distribution-Free estimation method demonstrated good and very good goodness of fit indices (*X*^2^/*df* = 1.54, GFI = 0.99, CFI = 0.94, TLI = 0.92, PGFI = 0.71, PCFI = 0.76, RMSEA = 0.03, and ECVI = 0.26). The known-groups method showed significant differences (*p* < 0.01) and effect sizes varying from small-to-medium to large. With respect to reliability, coefficients of 0.89 (CR) and 0.74 (KR20) for internal consistency and 0.85 for repeatability were found.

**Conclusion:**

The TCTOF-BRA presented satisfactory evidence, demonstrating it to be an instrument with valid and reliable measures for the evaluation of tactical knowledge (declarative and theoretical procedural), based on specific knowledge and decision making (cognitive domain), of Brazilian young soccer players from 12 to 17.9 years old.

## Introduction

Soccer (football) has been considered the most popular sport worldwide (Dvorak et al., 2004; FIFA, 2007; Shvili, 2020) and the tactics, either from an expanded and cognitive perspective (Abernethy et al., 1993; McPherson, 1994) or a dichotomous and ecological dynamics perspective (Silva et al., 2013), is recognized as the central dimension of the teaching-learning and training process (Teoldo et al., 2015; Praça and Greco, 2020), since it “gives meaning and consistency to all other dimensions” (Teoldo et al., 2015, p. 27). Systematic reviews have shown the relationship and influence of tactics, through the manipulation of small-sided games, in the technical, physical/physiological, and psychological dimensions of young soccer players (Sarmento et al., 2018; Bujalance-Moreno et al., 2019; Clemente and Sarmento, 2020). In this sense, the need for instruments that offer valid and reliable measures for the assessment of the tactical dimension is evident.

Studies and tests to assess the tactical domain of young soccer players are recent and few instruments meet the majority of quality criteria, as can be seen in the scoping review by Rechenchosky et al. (2021) and in the systematic review by Sánchez-López et al. (2021). Rechenchosky et al. (2021) further reveal that studies which developed and/or validated tests to assess the tactical dimension of young soccer players were mostly composed of young Europeans (75.0%), especially Spanish and Portuguese. Considering that “the population for which a test is intended should be clearly delimited” (American Educational Research Association [AERA] et al., 2014, p. 23), since the evidence of “validity and reliability are affected by the characteristics and composition of the sample” (Brink and Louw, 2012, p. 4), only five tests with participants from other continents were observed (Rechenchosky et al., 2021), including two with Brazilian samples, the TCTP-OE (Greco et al., 2015) and the TacticUP (Machado and da Costa, 2020).

The *Teste de Conhecimento Tático Processual para Orientação Esportiva (TCTP-OE)* is based on the “Game Test Situation” (Memmert and Roth, 2003), and evaluates tactical-technical behavior through a small-sided game of 3 × 3, being theoretically based on the general tactical principles (Garganta and Pinto, 1994). The TacticUP, on the other hand, is a test that assesses tactical knowledge through videos and is theoretically based on the core tactical principles of soccer (Costa et al., 2009). Therefore, the development, adaptation, or validation of instruments for assessing tactical knowledge in a Brazilian sample, using a questionnaire, with operational tactical principles (Bayer, 1994) as a theoretical basis, have not yet been verified.

Regarding the validation of instruments, according to Morales (2011, p. 3), “a test or a scale has as nature, the expression of the same trait” and the quality of the measure depends on the validation process. For Urbina (2014, p. 147), there is no consensus in the literature on which and how much validity evidence is needed for a given instrument to present a measure considered valid and reliable. For American Educational Research Association [AERA] et al. (2014, p. 11), “the process of validation involves accumulating relevant evidence to provide a sound scientific basis for the proposed score interpretations.”

In this sense, based on a series of references recognized in the scientific literature, Rechenchosky et al. (2021) proposed 13 criteria to be considered in instrument validation studies in the area of physical education and sport. One of the tests that meets a greater number of criteria (Rechenchosky et al., 2021; Sánchez-López et al., 2021), demonstrating more evidence in the validation process, is the *Test de Conocimiento Táctico Ofensivo en Fútbol (TCTOF)*, created and validated in Spain by Serra-Olivares and García-López (2016). The TCTOF is a questionnaire that assesses “declarative tactical knowledge” and “procedural tactical knowledge” in the cognitive/theoretical domain, considering the tactical dimension from an expanded and cognitive perspective (Abernethy et al., 1993; McPherson, 1994). Theoretical procedural knowledge represents knowledge in the representational plan (knowledge-based paradigm), and is related to what the player would do when faced with a hypothetical situation presented to them, for example, through videos and questionnaires (Rechenchosky et al., 2021). This is in line with Abernethy et al. (1993, p. 324) and McPherson (1994), when stating that the knowledge of “how to do” in sports of high strategy, as is the case of soccer, can refer to both the selection (cognitive) and the execution (motor) of the movement. For McPherson (1994, p. 230) “in sport a successful response selection (decision) may not necessarily correlate with successful response execution (action),” since a failure can be committed “due to an unsuccessful response execution, not response selection.” Thus, “an individual’s sport tactical knowledge may be confounded by the need to carry out a response selection in a sport situation.”

Therefore, considering the central importance that the tactical dimension assumes in the training and match process; the scarce availability of tests that assess the tactical dimension of young soccer players built or validated from Brazilian samples; the inexistence of instruments in this same population that have as a theoretical basis operational tactical principles and that are carried out using questionnaires, which tends to facilitate and expand their use by professors, coaches, and researchers; and also that the TCTOF involves decision making (cognitive domain) in game contexts, contributing to the ecological validity; it was chosen to “validate the *TCTOF* for the Portuguese language (Brazilian population)” based on the hypothesis that the TCTOF can offer valid and reliable measures for the assessment of tactical knowledge also in young male Brazilian soccer players. For this, two studies were organized with the following objectives: “Translate, adapt, and validate the content of the TCTOF-BRA” (Study 1) and “Determine and present the evidence of construct validity and reliability of the TCTOF-BRA” (Study 2).

## Study 1: Cross-Cultural Adaptation and Content/Face Validation

### Methods

#### Participants

A committee formed by the main researcher (LR), a doctoral student (VM), and a master’s student and soccer coach (MJ) participated in the translation, and received the support of the instrument’s main author (JS-O) and a postdoctoral professor (LB) in the Hispanic language. The “semantic analysis” (Pasquali, 2018, p. 107) of the preliminary version of the translated instrument was carried out by focus groups, using the “brainstorming” technique among researchers LR, VM, and MJ and 20 participants who represented the target sample (Under 13/U13, Under 15/U15, and Under 17/U17). The back translation was performed independently by two university professors, one in Brazil (PG) and another in Spain (DT), who did not participate in the translation, who have Spanish as their native language and proficiency in the Portuguese language (Beaton et al., 2000; Cassepp-Borges et al., 2010; International Test Commission, 2017). The cross-cultural equivalence stage (semantic, idiomatic, experiential, and conceptual) between the translated/pre-final version (Portuguese) and the original version (Spanish) involved the researchers who participated in the translation and back translation (VM, MJ, JS-O, PG, and DT), except LR and LB. For content validity, a panel of five university professors from the soccer area was formed, with at least 10 years of experience (Ericsson et al., 1993, p. 366) and who did not participate in any previous part of the research (AS, HS, JM, PB, and RA). Finally, complementing the content validity (American Educational Research Association [AERA] et al., 2014), for the face validity, a sample of 91 players aged 12.1 to 17.8 years (mean age ± SD = 15.1 ± 1.5 years) participated in a pilot study (pre-test), selected by convenience, who competed in the state championship and a regional championship. For both studies, it was decided to increase the age range of the sample in relation to that used in the development of the TCTOF, Spanish version (8–14 years of age). The minimum age of 12 years for the TCTOF-BRA was chosen after analysis by the committee of the Brazilian context and also following guidelines from the scientific literature (Brislin et al., 1973; Guillemin et al., 1993) regarding the age group for understanding translated questionnaires. It was also decided to apply the questionnaire to young people of 15, 16, and 17 years of age, since this is a basic category (U17), and to evaluate the behavior of the data/results in terms of difficulty and discrimination of each of the questions/items. Thus, the cross-cultural adaptation, content and face validity involved 12 researchers, including post-graduate students, coaches, and university professors, and 111 young male Brazilian soccer players.

#### Instrument

The *TCTOF* is a questionnaire with multiple-choice questions, involving statements and game contexts through pictures. It was created and validated in Spain by Serra-Olivares and García-López (2016) with the participation of 465 children and young people between 8 and 14 years old from different contexts. According to the authors, the test aims to assess tactical knowledge from a more ecological view and through two dimensions, the declarative and the procedural. The first contains 36 multiple-choice questions involving six indicators related to knowledge about: roles and positions, offside rule, individual technical-tactical elements, operational tactical principles (OTP), relationship between individual technical-tactical elements and OTP, and collective technical-tactical elements. The second contains 16 questions in the form of figures in which the participant must first choose “what” to do and then “how” to do it, related to the “why” do it (OTP) and involves four indicators related to decision making in situations of keeping/maintaining ball possession, advancing/progressing, and attacking/trying to score the goal, in addition to knowledge about the offside rule. Each correct answer has a value of 1 point and a higher score represents more tactical knowledge in soccer.

#### Procedures

First, the main author of the instrument was contacted in order to present interest and formally request authorization for the translation and adaptation of the test from Spanish to Portuguese, which was promptly answered. Subsequently, the project was submitted for ethical review, in accordance with the Declaration of Helsinki, and approved in March 2019 (CAAE 08918619.3.0000.0104; Opinion 3.208.874). For both studies, consent was obtained from the participants, their legal representatives, and the clubs and everyone’s privacy was preserved.

The method adopted for the cross-cultural adaptation of the instrument was back translation, associated with the committee method (Vallerand, 1989). Initially, the committee met (LR, VM, and MJ) to carry out the translation of the instrument from Spanish to Portuguese, as directed by the International Test Commission (2017), with regard to the committee being familiar with the test and taking due care regarding literal translations. Thus, the procedure involved the reading, discussion, and understanding of terms and denominations in Spanish based on Spanish references and the translation supported by Brazilian and Portuguese references (conceptual analysis). Terms that could raise doubts with respect to interpretation were registered for clarification with the other members of the committee (JS-O and LB). After a conversation between the main researcher (LR) and LB, a videoconference meeting (skype) was held between JS-O and LR, VM, and MJ, at which time all translated questions were presented. The main author of the instrument (JS-O) participated in the cross-cultural adaptation, clarifying doubts regarding the use of some terms, giving suggestions, and authorizing the changes proposed by the group.

Subsequently, theoretical/semantic analysis of the preliminary version, as an indicator of apparent validity, was performed by committee members and focus groups to verify if the items were clear and understandable. The literature suggests “3–4 participants per group” (Pasquali, 2018, p. 107). Thus, three groups (U13, *n* = 7; U15, *n* = 6; and U17, *n* = 7) with different levels of knowledge, according to their coaches, were constituted and independently asked if they understood the questions. Next, participants were asked about what each question sought to discover and what or how they would answer. In case of disagreement in the understanding between the participants and after suggestions, the question was rephrased and presented again to the youth players.

The next step was to send the version of the instrument translated to Portuguese to two professors who have Spanish as their native language to perform the back translation to Spanish. The two versions back translated to Spanish were sent to the main author of the instrument, who analyzed the questions and informed that they were preserved similar to the original instrument. According to Pacico (2015, p. 68), “many researchers ask the author of the original scale to evaluate the back translation”; if this is “similar to the original version (the meaning of the items is preserved) and the adapted items are adequate, data collection with the pilot sample can be started.” In sequence, after discussion and consensus, the committee (LR, VM, and MJ) consolidated the preliminary version of the instrument in Portuguese (pre-final version).

Although it was already possible to start the pre-test stage with the pilot sample, the authors chose to send the translated version (pre-final version) to the translators VM and MJ, to the retranslators PG and DT, and to the main author of the instrument (JS-O), to determine cross-cultural equivalence (semantic, idiomatic, experiential, and conceptual), according to Guillemin et al. (1993) and Beaton et al. (2000). The individuals were asked to analyze each question and select one of the options; 1 very poor equivalence; 2 poor equivalence; 3 average equivalence; 4 good equivalence; and 5 very good equivalence.

To investigate content validity, the panel consisting of five judges (AS, HS, JM, PB, and RA) received the translated version (pre-final) of the instrument and a spreadsheet on which they were required to score all questions/items in relation to the criteria using a Likert scale from 1 to 5 (very poor to very much): (a) Clarity of language: evaluates the terms and language used in the questions/items of the questionnaire, considering the characteristics of the target population; example: Do you believe the terms and language of the question are clear, understandable, and adequate for young soccer players (~12–17 years old)? How much?; (b) Practical relevance: assess the relevance of the question for the daily lives of the target population. This considers whether each question is designed to investigate the concept of interest and whether it happens in practice; example: Do you believe that the questions/situations are relevant to the practice of young soccer players? How much?; and (c) Theoretical relevance: assesses the degree of association between the question/item and the theoretical basis; analyzes whether the item is related to the construct that is intended to be measured; example: Do you believe that the content of this question/situation is relevant and representative of the knowledge you want to measure, or of one of its indicators, considering the construct in question (tactical knowledge)? How much?

**Figure 1:**
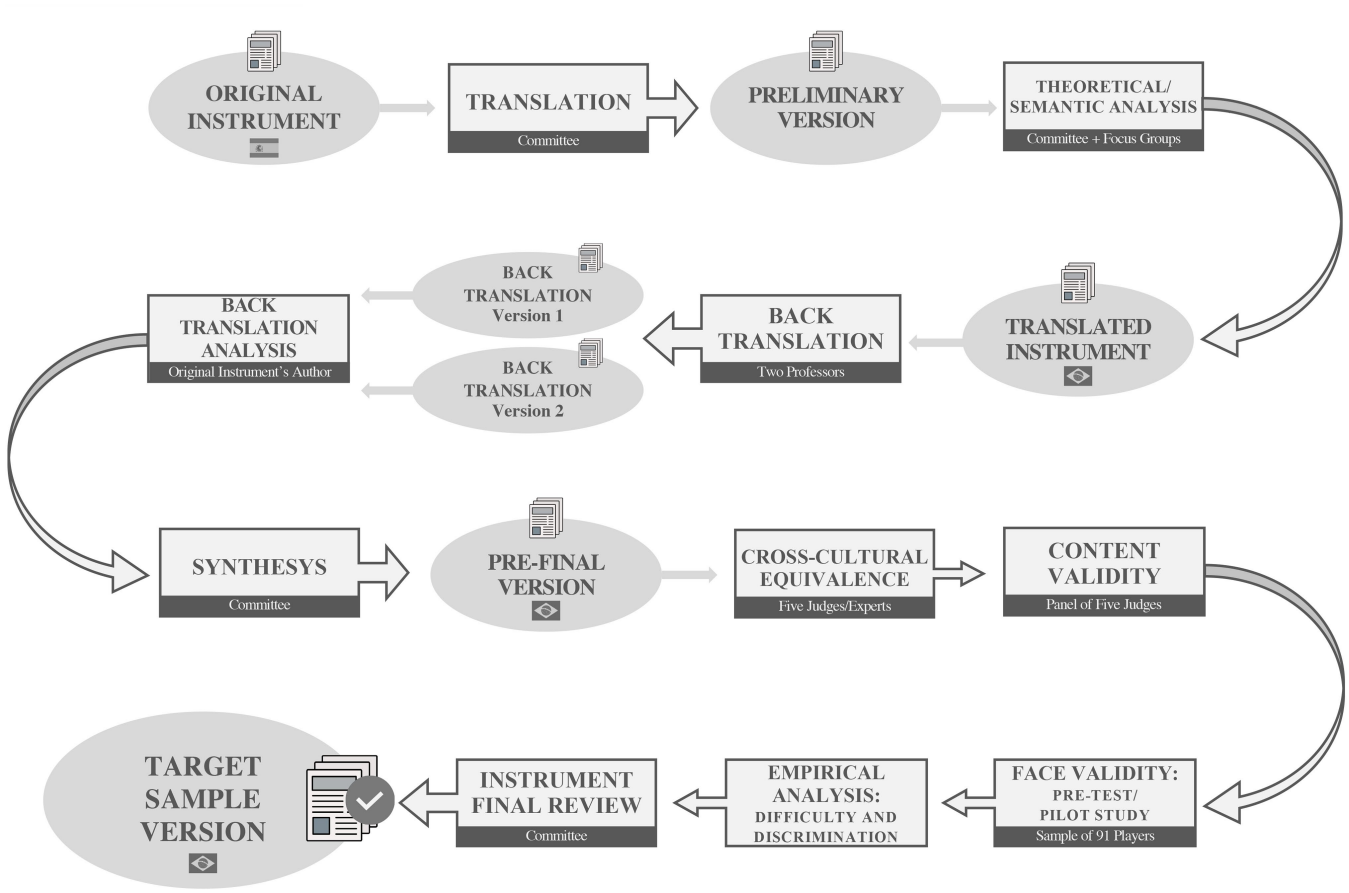
Study 1 flowchart. Source: the authors.

Subsequently, a pre-test (pilot study) was conducted to determine face validity, which “refers to the subjective judgment that participants make about the test” (Pacico and Hutz, 2015, p. 76) and indicates whether procedures are adequate and if any item remains incomprehensible. If the participant did not understand a question they were asked to circle it. At the end of the test, the youth players answered the following questions: (1) Do you think the test questions and figures are clear and understandable?; (2) Do you think this test assesses (tactical) knowledge about soccer?; (3) Did you enjoy taking the test?; (4) Did you feel challenged when taking the test?; and (5) Would you take the test at another opportunity to find out about your (tactical) knowledge in soccer, if necessary? All questions were initially answered with a yes or no; in case of “yes,” there was a Likert scale from 1 (very little) to 5 (very much). The pre-test data also allowed the “empirical analysis of the items” based on traditional parameters suggested in the literature, such as “difficulty and discrimination” (Pasquali, 2018, pp. 108–109). The summary of the procedures adopted in the cross-cultural adaptation and content/face validation is presented in Figure 1.

#### Data Analysis

The agreement regarding cross-cultural equivalence and content validity was determined by the Coefficient of Content Validity (CCV) proposed by Hernández-Nieto (2002, p. 131-132), which is able to measure the degree of agreement between judges regarding the total for the instrument, total per equivalence/parameter, and total per question (CCV_t_), as well as considering each item/question per type of equivalence/parameter (CCV_i_). If any question is considered unsatisfactory, it must be adjusted before the questionnaire is applied to the target population. Hernández-Nieto recommends a cutoff value of 0.80. Balbinotti (2004), on the other hand, suggests that it is possible to consider a CCV between 0.70 and 0.79 as the threshold and less than 0.70 as unsatisfactory. Therefore, in this study, a value of 0.70 was adopted as the threshold for the assessment of cross-cultural equivalence and content validity.

Face validity and the number of questions not understood were obtained by relative frequency (%). The empirical analysis of the items involved the difficulty index (number of subjects who answered the item correctly/total number of subjects who answered the item), discrimination index D (Flanagan method), and item-total point-biserial correlation. Difficulty indices from 0.10 to 0.90 and discrimination indices *D* ≥ 20 (0.20) were sought (Thomas et al., 2015, p. 396) and item-total point-biserial correlation coefficients ≥0.30 (Field, 2009, p. 598; Pasquali, 2018, p. 136).

### Results

All questions from the pre-final version applied in the pilot study are presented in Supplementary Table 1 (Data Sheet 1).

In the cross-cultural evaluation, the CCV_t_ of the instrument was 0.96, with a semantic equivalence of 0.96, idiomatic equivalence of 0.95, experiential equivalence of 0.96, and conceptual equivalence of 0.96. The CCV_t_ per question (average of equivalences) ranged from 0.80 to 1.00 (see Supplementary Table 2 in the Data Sheet 1). Considering CCV by question and type of equivalence (CCV_i_), all values were ≥ 0.80, with the exception of the conceptual equivalence of the questions about “offside position” and “permute,” which had a CCV_i_ considered as threshold (0.76). Therefore, the cross-cultural evaluation showed semantic, idiomatic, experiential, and conceptual equivalence for all questions. The *Test de Conocimiento Táctico Ofensivo en Fútbol (TCTOF)* was named in Portuguese as the *Teste de Conhecimento Tático Ofensivo no Futebol (TCTOF-BRA)*.

In content validity, the CCV_t_ of the test was 0.87, with 0.87 for clarity of language (CL), 0.86 for practical relevance (PR), and 0.87 for theoretical relevance (TR). The CCV_t_ per question (average of criteria) ranged from 0.63 to 1.00. The CCV_i_ was below the threshold (0.70) for CL in questions 1 (player’s role while attacking) and 33 (permute), for PR in questions 2–5 (role and positions), and for TR in question 23 (controlling × OTP). Question 27 about “Give-and-go” or “wall pass” had CCV_i_ < 0.70 in CL, PR, and TR. Considering the relevance of reaching the threshold value in all criteria (CL, PR, and TR), eight questions (1, 2, 3, 4, 5, 23, 27, and 33) had at least one value below 0.70 (see content validity column in Supplementary Table 2 of the Data Sheet 1). Thus, 43 (84.3%) questions demonstrated content validity.

Face validity showed that more than 95.5% of the sample found the questions clear and understandable, thought that the test assesses tactical knowledge in soccer, enjoyed taking the test, and would do it again, if necessary. Approximately, 2 out of every 10 participants evaluated (21.3%) declared that they did not feel challenged when taking the test. Finally, in only four questions (1, 16, 27, and 33) more than 10% of the participants declared that they did not understand the question or a part of it. One question (15) received 8.8% and all the others demonstrated values below 5%. The average application time was 32.9 ± 8.4 min (SD). Finally, an empirical analysis of the difficulty (DI) and item discrimination (*D* and *R*_pb_) was performed after applying the pilot instrument (see Supplementary Table 1 in the Data Sheet 1). It is possible to verify (in bold) that 26 questions together met the criteria in the three parameters (DI ≥ 0.10 and ≤ 0.90; *D* ≥ 20 (0.20); and *R*_pb_: ≥ 0.30).

### Discussion

In order for an instrument to be used with subjects from a different country to the one for which it was created and validated, it must be submitted to a “systematic” process of translation and validation (Cassepp-Borges et al., 2010, p. 508).

Vallerand (1989) indicates that the first moment of translation, which in the case of the present study is transferring the Spanish version into Portuguese, can be performed by a single individual (traditional translation), while Cassepp-Borges et al. (2010) suggest one or more independent translations. Furthermore, Vallerand suggests the use of a committee to avoid possible prejudices of a single individual and, if possible, the involvement of the person who created the instrument. Beaton et al. (2000) also argue the importance of participation of the instrument’s author, since he/she can assess more complex situations and suggest terms that demonstrate content validity in both languages. Sometimes translators with proven competence in the languages are hired, but who do not have knowledge of the study’s object, which can compromise or hinder the next steps. In this sense, the International Test Commission (2017, p. 11) advises that the translators or team are familiar with the test. For these reasons, in some studies, the authors themselves carried out the translation and adaptation, as was the case in Maroco et al. (2008). Therefore, the translation in this study followed guidance from important references in the area, aiming to ensure quality in this and other stages.

After the translation stage, characterized in the current study, by translation (Spanish—Portuguese), theoretical/semantic analysis (focus groups), and back translation (Portuguese—Spanish), the cross-cultural equivalence between the translated version and the original version was determined. Based on the CCV results, it is possible to state that the questions in the Spanish and Portuguese versions have the same meaning (semantic equivalence), the expressions are equivalent, that is, there is no change in cultural meaning (idiomatic equivalence), the content is present in both realities (experiential equivalence), and the questions are conceptually equivalent, that is, they assess the same aspect in different cultures (conceptual equivalence). Several authors have addressed the issue of cross-cultural adaptation and equivalences are well described in Guillemin et al. (1993) and in Beaton et al. (2000). It is important to highlight that substitution with another term to preserve the desired equivalence is allowed and, therefore, was performed when necessary. Additionally, sometimes a given situation may simply not be performed (even if it is translatable) in another country or culture, in this case the questionnaire item needs to be replaced by a similar item or even excluded (Guillemin et al., 1993). This situation occurred in the item about offside position, which had to be almost completely changed, and in item 23 of the original questionnaire, which was excluded because in Portuguese it was the same technical skill (shooting), as previously presented in another item of the instrument.

Finally, according to Guillemin et al. (1993), in the stage of cross-cultural analysis, one of the committee’s functions is to modify instructions or formats, modify or reject inappropriate items, and generate new items; ultimately, it is likely that the committee modify or eliminate irrelevant, inappropriate, or ambiguous items and may generate substitutes that better fit the target culture while maintaining the overall concept of the excluded items. Part of this committee’s role should also be to review the introduction and instructions for completing the questionnaire. Thus, adjustments were made to the questionnaire after focus groups, contact with the main author of the instrument, and discussion by the committee. Based on the results found, the cross-cultural assessment showed semantic, idiomatic, experiential, and conceptual equivalence between the Spanish instrument and the one translated to Brazilian Portuguese, that is, there was correspondence between the questions of the original TCTOF and the TCTOF-BRA.

Content validity involves evidence of the extent to which the test or instrument represents the content or behavior that is intended to be measured (Goodwin and Leech, 2003; Pasquali, 2010). For American Educational Research Association [AERA] et al. (2014, p. 14), the “evidence based on test content” involves an “analysis of the relationship between the content of a test and the construct.” The main method used is agreement between an expert panel. Hernández-Nieto (2002, p. 119) recommends a “minimum of three and a maximum of five judges,” preferably per modality, and, according to Balbinotti et al. (2007, p. 32), who “did not participate in any stage of the study.” In this study, five university professor judges were involved, with experience ranging from initiation to high performance in soccer. Regarding the cutoff points, the value of 0.70 was chosen as the threshold, according to Balbinotti (2004) and following validation studies in the area (Silva, 2018) and outside it (Balbinotti et al., 2007). For Cassepp-Borges et al. (2010, p. 513) it is possible to “relativize the cutoff point” due to the different opinions among the judges. In addition, it is worth highlighting that seven experts had already validated the original version of the TCTOF in terms of content (Serra-Olivares and García-López, 2016).

As seen, the CCV_t_ results for the test showed satisfactory agreement and content validity (Hernández-Nieto, 2002). The clarity of language was confirmed, that is, the terms and language of the questions are clear, understandable, and adequate for young male soccer players between 12 and 17.9 years of age; the judges also considered that there is practical relevance, that is, the questions and game situations presented in the figures are related to the daily lives of young soccer players; and there is theoretical relevance, that is, the content of the questions is relevant and representative of the knowledge that is being measured or of one of its indicators (Cassepp-Borges et al., 2010).

After the return of the content validity evaluation form completed by the judges, the committee met to discuss the results and the observations and suggestions made, especially in the questions with the lowest scores. Thus, 14 questions were reformulated according to the experts’ assessment before applying the questionnaire to the pilot sample (pre-test). Some authors (Greco et al., 2015) have suggested excluding items that are assessed as having a practical relevance below the cutoff point adopted, as they would not be considered relevant to the reality of the target population. However, it was decided not to remove any question in the initial stages of the study and analyze how their behavior after the application in the population of interest. In short, 43 (84.3%) questions showed content validity and this was also the number of questions that together had satisfactory results both in the cross-cultural adaptation and in content validity, since in the first, all items reached the necessary equivalences.

Regarding face validity, this type of validity generally receives less attention from researchers, as seen in Rechenchosky et al. (2021). According to Bornstein (1996), this validity can impact other forms of validity, hence the need to include it as one of the stages in the instrument validation process, beginning with the pre-test or pilot study (Guillemin et al., 1993). Thus, in order to ensure that the translation was understandable, this stage intended to identify questions that were not clear to the participants. The subjects of the pilot sample were asked to circle the questions they did not understand as a whole or in part; this procedure was also adopted in the original TCTOF study. The literature suggests that language should be understood by children aged 10–12 years old (Brislin et al., 1973; Guillemin et al., 1993). For the authors of this study, the few questions (1, 16, 27, and 33) that received a higher percentage (10–20%) of not being understood were within the expected range; even so, they were adjusted for the application of the instrument to the target sample.

Although the literature indicates that the sample size in the pilot study (pre-test) does not need to be greater than 10% of the target sample (Canhota, 2008, p. 70), in this study, it was approximately 12%. For statistical analysis this quantity is insufficient (Morales, 2011, p. 47), as it would be ineffective for these cases, however, it is believed that the pilot study, in addition to enabling apparent validity, can provide valuable information regarding the difficulty of the questions, which in turn can affect discrimination indices and, later, other evidence of validity. Knowing the behavior of these variables enables immediate adjustments to the instrument for application in the target sample.

Regarding the cutoff points adopted to analyze the difficulty and discrimination of the questions, some are more conservative than those adopted in this study, as is the case of Nakano et al. (2015), who consider a good difficulty index between 0.30 and 0.70; others are less conservative and justify it by the sample size, that is, for the analysis of discrimination, for example, in the case of large samples, correlation coefficients lower than 0.30 would already be “acceptable” (Field, 2009, p. 598). Regarding the *D* index, fundamentally, what is sought is for it to be positive and distant from zero, since “a null or negative *D* demonstrates that the item is not discriminatory” (Pasquali, 2018, p. 133).

Based on the knowledge that “the pilot study is important as a last chance to detect and correct errors before carrying out the research” (Cassepp-Borges et al., 2010, p. 514), a new committee meeting was held to analyze and discuss the results, and review (Guillemin et al., 1993) and make final adjustments to the instrument, preserving the logic of the questions and answers.

Thus, considering the cross-cultural adaptation, the evidence of content and face validity, and the limitations for a more appropriate analysis of the behavior of the questions regarding difficulty and discrimination, due to the sample size, it was decided to maintain the 51 questions for the final data collection (target sample), although it was possible to conclude satisfactory evidence in 43 questions of the TCTOF for application in Brazil. According to Cassepp-Borges et al. (2010, p. 513), when a question “is not considered relevant to the reality” that is being sought, it is possible for it to remain in the questionnaire, since “the researcher may insist on establishing some comparability.” Finally, in response to these authors (Cassepp-Borges et al., 2010, p. 519), Study 2 sought a balance between improving the structure of the instrument (necessary) and keeping it similar to the original.

## Study 2: Evidence of Construct Validity and Reliability of the TCTOF-BRA

### Methods

#### Participants

The sample consisted of 768 young male soccer players between 12.0 and 17.9 years of age (mean age ± SD = 15.0 ± 1.5 years) from 24 Brazilian states and five regions of the country. The players belonged to eight clubs that competed in the Paraná (state) championship, in addition to national and international tournaments, and eight clubs that mainly competed in regional championships. The average time of soccer practice was 6.6 ± 2.6 (SD) years and the average number of training sessions per week was 4 days (88% ≥ 3 × per week). Considering color or ethnicity (Dias et al., 2009), 42.3% recognize themselves or identify themselves as white (Caucasian descent), 38.8% as mixed race, 13% as black (African descent), 3.3% as indigenous, and 2.6% as yellow (Asian descent). Initially 796 youth players completed the questionnaire, but 28 were excluded from the final composition of the sample due to the following criteria: 20 for being under 12 years old, one over 18 years old, and seven for incorrect filling out or lack of information that would compromise data analysis. Therefore, 205 male players from the Under 13/U13 category, 340 from the Under 15/U15 category, and 223 from the Under 17/U17 category participated in this stage. The temporal stability of the TCTOF-BRA was verified based on 85 players (mean age ± SD = 14.3 ± 1.6 years old) who repeated the test.

#### Procedures

Data collection was carried out from November 04, 2019 to December 06, 2019 by the main researcher of the study, with the assistance of a doctoral student and, in some situations, also a master’s student and soccer coach. Collections were carried out by category, with groups of approximately 20–30 players. A room with chairs, clipboards, questionnaires, and pens was prepared in advance. At the beginning of collection the team and study objectives were always presented, followed by the guidance and completion of the first part of the instrument. After everyone had concluded this part, the guidance and completion of the second part occurred. Face/apparent validity was also determined in the target sample through five questions answered at the end of the test.

For the “selection of questions” that were included in the factor analysis (Field, 2009, p. 572), the “theoretical analysis” (cross-cultural adaptation and content validity) and “empirical analysis of the items” (difficulty, discrimination, and unidimensionality) were used, according to Pasquali (2018, p. 106), as well as by analysis of agreement (kappa). This procedure is also in line with Thomas et al. (2015, p. 395), when approaching the “analysis of items in knowledge tests as a way to determine which questions are suitable and which need to be rewritten or discarded.” As the sample size is now sufficient for statistical analyses, it was decided to examine all the questions of the instrument, not only the 43 that demonstrated content validity in Study 1. After EFA and CFA, it was verified whether the fit of the modified model was significantly better than that of the original model. To verify the temporal stability of the TCTOF-BRA, 11.1% of the sample completed the instrument again after the first application (mean ± SD = 9.5 ± 3.2 days).

#### Data Analysis

Face validity was determined by relative frequency (%). In the theoretical analysis of the items, a CCV of 0.70 was adopted as the threshold for cross-cultural adaptation and content validity. In the empirical analysis, the parameters and cutoff points were: difficulty index (DI) ≥ 0.10 and ≤ 0.90; discrimination index (*D*) ≥ 20 (0.20; Thomas et al., 2015, p. 396), item-total point-biserial correlation (*R*_pb_) ≥ 0.30 (Field, 2009, p. 598; Pasquali, 2018, p. 136); factor loading ≥0.30 (Hair et al., 2014, p. 115); and kappa agreement >0.20, *p* < 0.05 (Landis and Koch, 1977).

Exploratory factor analysis (EFA) was used to assess the factor structure of the TCTOF-BRA, using Factor Analysis software v.10.10.03 (Lorenzo-Seva and Ferrando, 2020) from the tetrachoric correlation matrix (Maroco, 2007, p. 406; Marôco, 2014, p. 227), according to the dichotomous/nominal nature of the variables, with factor extraction by the Robust Diagonally Weighted Least Squares (RDWLS) method (Asparouhov and Muthén, 2010) and Robust Promin oblique rotation (Lorenzo-Seva and Ferrando, 2019). To define the number of retained factors, the Kaiser (eigenvalue > 1) and Cattel (scree plot analysis) criteria were used, as well as the theoretical basis of the test. The multidimensionality of the instrument was investigated by “factor loadings” (Pasquali, 2018, p. 117) and by the indicators Unidimensional Congruence (UniCo < 0.95) and Explained Common Variance (ECV < 0.85) (Ferrando and Lorenzo-Seva, 2018). The EFA validity was evaluated by the Kaiser–Meyer–Olkin (KMO) measure of sampling adequacy, in which values ≥0.50 are considered acceptable (Maroco, 2007, p. 368; Pestana and Gageiro, 2005, p. 491) and by the Bartlett’s Test of Sphericity, in which *p* < 0.05 is expected. Multicollinearity was also analyzed by the matrix determinant, which must present a value greater than 0.00001 (Field, 2009, p. 581). The adequacy of the model in the EFA was evaluated using the fit indices *X*^2^/gl, Goodness of Fit Index (GFI), Comparative Fit Index (CFI), Tucker–Lewis Index (TLI), and Root Mean Square Error for Approximation (RMSEA), according to the reference values presented in Supplementary Frame 1 (Data Sheet 1).

Confirmatory factor analysis (CFA) was performed by the Asymptotically Distribution-Free (ADF) estimation method and by the Robust Diagonally Weighted Least Squares (RDWLS) in SPSS Amos and JASP software, respectively. Possible outliers were evaluated using the *Mahalanobis* distance and the assumption of normality was verified by asymmetry (*sk* < 3), kurtosis (*ku* < 10) (Kline, 2016, pp. 76–77), and multivariate kurtosis (*ku* < 10). The quality of the goodness of fit was determined based on the indices and reference values presented in Supplementary Frame 1 in Data Sheet 1 (Marôco, 2010, p. 51), and the adjustment was made based on the modification indices (> 11; *p* < 0.001), according to Marôco (2010, p. 54). The equations were obtained following the values presented in the weight matrix using AMOS software.

Construct validity was also verified by the known-groups difference method, from the comparison between categories (age) and between the expertise, using the Mann–Whitney U test (*p* < 0.01). Estimates of effect size (ES) were initially obtained using an equation for nonparametric data ( $r=z/\sqrt{N}$ ), where *r* is the point-biserial correlation, and transformed into Cohen’s *d* using the formula $d=2r/\sqrt{\left(1-r^{2}\right)}$ , according to Fritz et al. (2012, pp. 12, 9) and Ivarsson et al. (2013, p. 99). The CIs for effect sizes (95%) were calculated with the formula CI=ES−1.96setoES+1.96se (Nakagawa and Cuthill, 2007) where *se* is the asymptotic standard error of the effect size. The *se* value was calculated from Cohen’s *d*, with the following equation: $se\;\left(d\right)=\sqrt{\left(n1+n2-1)/(n1+n2-3\right)}\left[\left(4/\left(n1+n2\right)\right)\left(1+d^{2}/8\right)\right]$ (Ivarsson et al., 2013). ES values were classified based on Cohen (1988) as small (*d* ≤ 0.20), small-to-medium (0.21 ≤ *d* ≤ 0.50), medium-to-large (0.51 ≤ *d* ≤ 0.80), or large (*d* ≥ 0.81).

The internal consistency of the TCTOF-BRA was determined by composite reliability, through CFA results (>0.60; Bagozzi and Yi, 1988) and by the Kuder–Richardson coefficient (KR20), a special case of Cronbach’s Alpha used for dichotomous variables; the reference values adopted for KR20 were those of Landis and Koch (1977). The repeatability was analyzed by the test–retest agreement through the Intraclass Correlation Coefficient, also adopting the cutoff points of Landis and Koch (1977): <0.20 slight agreement, 0.21–0.40 fair, 0.41–0.60 moderate, 0.61–0.80 substantial, and 0.81–1.00 almost perfect agreement.

### Results

The face validity was confirmed in the target sample (*n* = 768) with more than 96% of participants stating that the questions are clear and understandable (98.7%), that the test assesses tactical knowledge in soccer (99.6%), that they enjoyed it (97.9%), and would do it again (96.9%), if necessary. For every 10 participants evaluated, around eight considered the test challenging (79.3%).

For better analysis of the information obtained to date, Supplementary Table 2 (Data Sheet 1) shows the main results of the cross-cultural adaptation (CCV_t_ per question) and an overview of the content validity (CCV_i_) for Study 1, in addition to the percentage of ununderstood questions, difficulty, discrimination, unidimensionality, and agreement indices obtained in Study 2. The eight questions that did not reach the content validity in Study 1 (1, 2, 3, 4, 5, 23, 27, and 33) also did not show satisfactory results in the target sample, although questions 1 and 33 demonstrated problems practically only with regard to the lack of item understanding (10.8 and 13.8%, respectively), confirming the insufficient clarity of language pointed out by the experts (Study 1).

Of the 51 questions, 16 met the criteria established for inclusion in the EFA and are highlighted in gray in Supplementary Table 2 (Data Sheet 1). Due to the substantial agreement (kappa = 0.66), factor loading >0.30, discrimination indices close to the cutoff points, and because it is an important question for understanding the logic and theoretical basis of the instrument, it was decided to include question 17 on operational tactical principles (OTP): keep/maintain possession of the ball. Although questions 21 and 24 achieved satisfactory results, when performing a first EFA, they were saturated in different factors. In addition, in the first meetings with the main author of the instrument, he suggested the exclusion of the questions that associate the individual technical-tactical elements with the OTP (20–26) due to their complexity and because they have a different pattern of answers from the rest of the instrument (one, two, or three correct alternatives), thus, it was decided to exclude these two questions, leaving the TCTOF-BRA with 15 items to be submitted to the EFA and CFA.

The EFA showed a model with adequate fit (KMO = 0.69 [0.67–0.77]; Bartlett *p* < 0.001) composed of four factors that explain 58.2% of the total variance of the data (Table 1). The unidimensionality indicators (Unidimensional congruence—UniCo = 0.912 and Explained common variance—ECV = 0.757) confirmed the assumption that the TCTOF-BRA is a multidimensional instrument. The determinant was 0.002 and no pattern of cross-loadings was found, that is, items with factor loadings ≥0.30 in more than one factor. The goodness of fit indices showed a factor structure considered very good: *X*^2^/df = 1.53, GFI = 1.00, CFI = 0.992, TLI = 0.984, and RMSEA = 0.026.

**Table 1:** Exploratory factor analysis (EFA) of the four factor model TCTOF-BRA.

Questions	Factor 1	Factor 2	Factor 3	Factor 4	
2. What do you do if you are the attacking player with the ball? (Attack)	0.50			
4. What do you do if you are the attacking player with the ball? (Progress)	0.75			
6. What do you do if you are the gray player who does NOT have the ball? (Maintain)	0.26			
10. What do you do if you are the attacking player with the ball? (Progress)	0.91			
12. What do you do if you are the attacking player with the ball? (Progress)	0.43			
15. What do you do if you are the gray player who does NOT have the ball? (Progress)	0.59			
17) What do you understand about keeping ball possession?		0.36		
18) What do you understand about moving towards the opponent’s goal?		1.03		
19) What do you understand about attacking the opponent’s goal?		0.92		
28) Providing “width” in the attack is?			0.80	
30) Providing “depth” in the attack is?			0.79	
31) Creating numeric superiority situations in attack are:			0.29	
32) Creating free spaces are:			0.25	
6) In soccer, a player is in offside position when:				0.76
16. In which images would you be in offside position?				0.67
Percentage of the total variance explained (%)	32.3	10.5	8.2	7.2

Factor 1: decision making; Factor 2: operational tactical principles; Factor 3: collective tactical-technical elements; Factor 4: offside rule; EFA performed from the tetrachoric correlation matrix; Extraction method: Robust Diagonally Weighted Least Squares (RDWLS); Rotation method: Robust Promin. The item number format follows the original instrument and was preserved to identify which questions of the TCTOF, Spanish version, were included in the Brazilian version (TCTOF-BRA); the “NUMBER)” format refers to Part 1 questions and the “NUMBER.” format refers to questions of the figure type: example “6)” and “6.,” respectively.

As for normality of data, all 15 questions showed asymmetry <3 and 14 of them kurtosis <10. As one question showed kurtosis of 15.3 and the multivariate kurtosis of 45.7, the Asymptotically Distribution-Free (ADF) estimation method was chosen for CFA. According to Marôco (2010, p. 203), when the variables are not quantitative (continuous), “the fit of the model must be carried out with methods that do not require the assumption of normality, as is the case of the ADF method.” Additionally, and to confirm the estimates found, the Robust Diagonally Weighted Least Squares (RDWLS) method was also applied. Possible outliers did not affect the goodness of fit.

**Figure 2:**
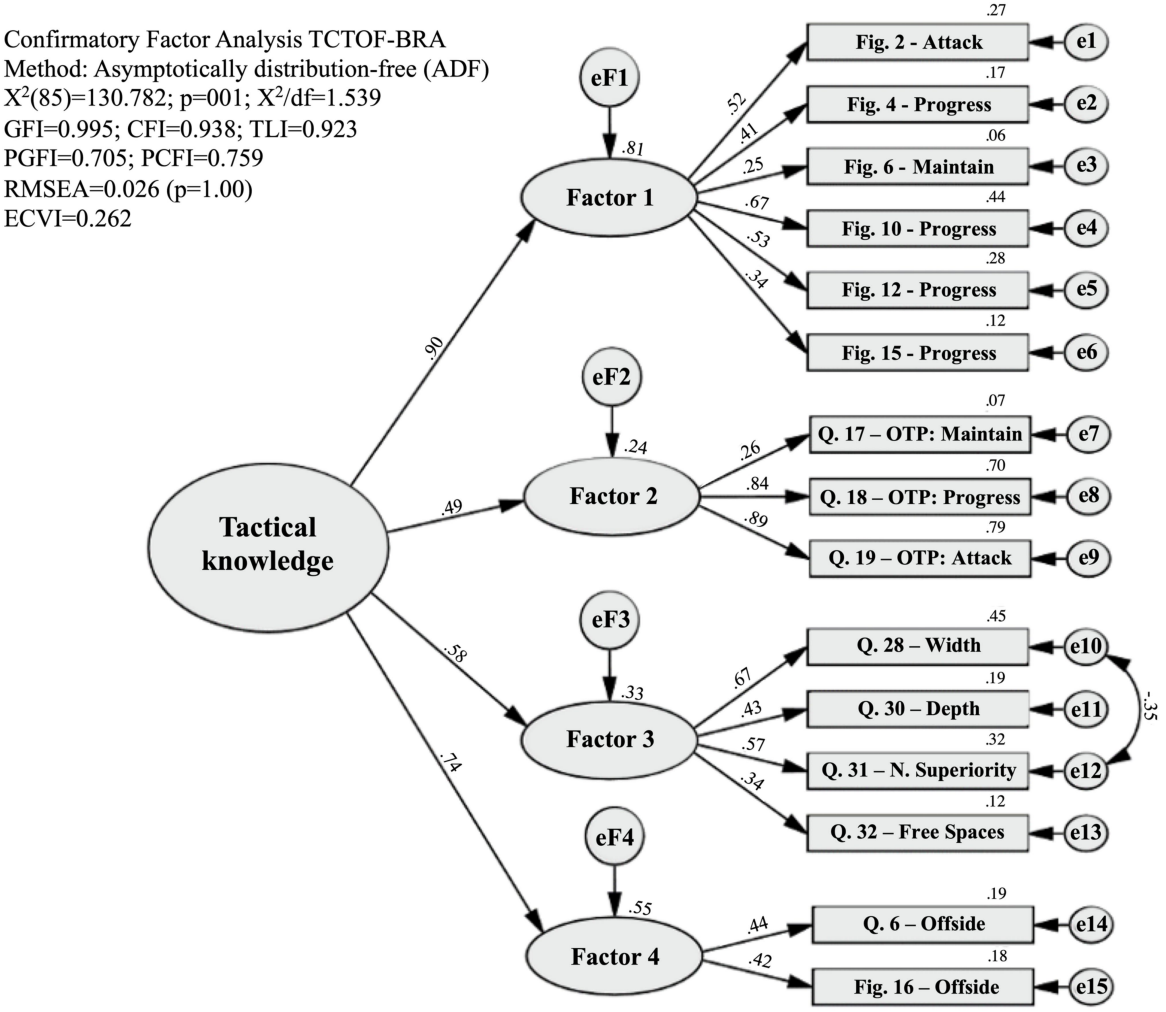
Second-order CFA (Model 3). Source: the authors.

The results of the CFA using the ADF method are shown in Table 2, according to the tested models. The final model with the 2nd order factor analysis is shown in Figure 2. Please see the Data Sheet 1 for Supplementary Figures 1, 2 referring to models 1 and 2.

**Table 2:** Confirmatory factor analysis (CFA) of the TCTOF-BRA: ADF method.

Model	df	*X* ^2^	*p*	*X*^2^/df < 2	GFI > 0.90	CFI > 0.90	TLI > 0.90	PGFI > 0.60	PCFI > 0.60	RMSEA < 0.1	*p* ≥ 0.05	ECVI
1	84	151.508	0.000	1.804	0.994	0.973	0.885	0.696	0.726	0.024	1.00	0.252
2	83	129.549	0.001	1.561	0.995	0.937	0.920	0.688	0.740	0.027	1.00	0.265
3	85	130.782	0.001	1.539	0.995	0.938	0.923	0.705	0.759	0.026	1.00	0.262

df, degrees of freedom; *p*, *p* value; *X*^2^, Chi-square; GFI, goodness of fit index; CFI, comparative fit index; TLI, Tucker–Lewis index; PGFI, parsimony goodness of fit index; PCFI, parsimony comparative fit index; RMSEA, root mean square error of approximation; ECVI, expected cross-validation index. Model 1: First-order factor analysis, according to EFA results. Model 2: Correlation between the errors in questions “width and numeric superiority.” Model 3: Second-order factor analysis.

Although the three models showed very good or good fit indices, the modified second order model (Model 3) was significantly better (*p* < 0.001) than the original model $\left(X_{dif}^{2}=20.7>X_{0.999;\left(1\right)}^{2}=10.8\right)$ , rejecting the null hypothesis that the models have the same goodness of fit, which indicates greater factor validity (Marôco, 2010, p. 187).

The CFA results by the RDWLS method are shown in Table 3 and were even better than those found by the ADF estimation method.

**Table 3:** CFA of the TCTOF-BRA: RDWLS method.

Model	df	*X* ^2^	*p*	*X*^2^/*df* < 2	GFI > 0.90	CFI > 0.90	TLI > 0.90	PGFI > 0.60	PCFI > 0.60	RMSEA < 0.1	*p* ≥ 0.05	ECVI
1	84	98.638	0.131	1.174	0.999	0.992	0.990	0.799	0.794	0.015	1.00	0.262
2	82	61.743	0.954	0.753	0.999	1.000	1.014	0.780	0.781	0.000	1.00	0.219
3	84	64.527	0.943	0.768	0.999	1.000	1.013	0.799	0.800	0.000	1.00	0.217

df, degrees of freedom; *p*, *p* value; *X*^2^, Chi-square; GFI, goodness of fit index; CFI, comparative fit index; TLI, Tucker–Lewis index; PGFI, parsimony goodness of fit index; PCFI, parsimony comparative fit index; RMSEA, root mean square error of approximation; and ECVI, expected cross-validation index. Model 1: First-order factor analysis, according to EFA results. Model 2: Correlation between the errors in questions “width and depth” and “OTP: progress and attack.” Model 3: Second-order factor analysis.

The second order hierarchical structure, according to Maroco et al. (2008, p. 641), “allows estimation of a total score” of the latent variable, in this case, of tactical knowledge. Using the values presented in the weight matrix (CFA output), the equations for the overall score of the TCTOF-BRA (tactical knowledge) and for each of the factors are described in Supplementary Frame 2 (see Data Sheet 1), according to Marôco (2010, p. 200).

The maximum values that can be obtained, when the participant correctly marks the 15 questions, are: tactical knowledge = 3.774; factor 1 = 0.741; factor 2 = 0.132; factor 3 = 0.917; and factor 4 = 0.526. So that the overall and by factor scores can be better understood and interpreted without any harm to the scores to be obtained, the maximum values were activated at the end of the equations as constants so that the maximum score is 10.0 points (Frame 1).

**Frame 1:** Equations to estimate tactical knowledge and factors 1, 2, 3, and 4 with addition of constants.

Variable	Equation
Tactical Knowledge	(0.045xQ_1_ + 0.214xQ_2_ + 0.301xQ_3_ + 0.337xQ_4_ + 0.083xQ_5_ + 0.255xQ_6_ + 0.07xQ_7_ + 0.315xQ_8_ + 0.403xQ_9_ + 0.295xQ_10_ + 0.125xQ_11_ + 0.559xQ_12_ + 0.341xQ_13_ + 0.179xQ_14_ + 0.252xQ_15_) x (10/3.774)
Factor 1	(0.006xQ_1_ + 0.029xQ_2_ + 0.04xQ_3_ + 0.045xQ_4_ + 0.011xQ_5_ + 0.034xQ_6_ + 0.009xQ_7_ + 0.042xQ_8_ + 0.104xQ_9_ + 0.076xQ_10_ + 0.032xQ_11_ + 0.145xQ_12_ + 0.088xQ_13_ + 0.046xQ_14_ + 0.034xQ_15_) x (10/0.741)
Factor 2	(0.01xQ_1_ + 0.046xQ_2_ + 0.064xQ_3_ + 0.001xQ_4_ + 0.001xQ_6_ + 0.001xQ_8_ + 0.002xQ_9_ + 0.001xQ_10_ + 0.001xQ_11_ + 0.002xQ_12_ + 0.001xQ_13_ + 0.001xQ_14_ + 0.001xQ_15_) x (10/0.132)
Factor 3	(0.003xQ_1_ + 0.013xQ_2_ + 0.018xQ_3_ + 0.331xQ_4_ + 0.082xQ_5_ + 0.25xQ_6_ + 0.069xQ_7_ + 0.019xQ_8_ + 0.025xQ_9_ + 0.018xQ_10_ + 0.008xQ_11_ + 0.034xQ_12_ + 0.021xQ_13_ + 0.011xQ_14_ + 0.015xQ_15_) x (10/0.917)
Factor 4	(0.004xQ_1_ + 0.021xQ_2_ + 0.029xQ_3_ + 0.033xQ_4_ + 0.008xQ_5_ + 0.025xQ_6_ + 0.007xQ_7_ + 0.119xQ_8_ + 0.039xQ_9_ + 0.029xQ_10_ + 0.012xQ_11_ + 0.055xQ_12_ + 0.033xQ_13_ + 0.017xQ_14_ + 0.095xQ_15_) x (10/0.526)

Factor 1, decision making; Factor 2, operational tactical principles; Factor 3, collective tactical-technical elements; Factor 4, offside rule; Q, TCTOF-BRA questions. For every correct question, *Q* = 1; constants obtained from factor score weights calculated by AMOS; additional constant added to the end of the equation for a maximum value of 10.0 points in tactical knowledge or analyzed factor.

The known-groups difference method, as further evidence of construct validity, showed a significant difference in the scores of factors 1, 3, and 4 and in the overall result of the TCTOF-BRA among all categories (age), in favor of the oldest players; in factor 2 there was only no difference between U13 and U15, although the effect size was 0.31 (small-to-medium). As for expertise, the results were significantly higher in all factors and in the overall result in favor of those who compete in the state championship or higher. This information and the effect sizes are shown in Table 4.

**Table 4:** Construct validity: known-groups difference method.

	TCTOF-BRA (*n* = 768)				
	Factor 1	Factor 2	Factor 3	Factor 4	TCTOF-BRA overall
	P25 P50 P75				
Category	(Max. 10.0 pts)				
U17 _(*n* = 223)_	6.56 8.38 9.31	9.39 9.77 9.92	7.78 9.12 9.77	7.28 8.69 9.45	6.90 8.40 9.20
U15 _(*n* = 340)_	5.35 7.34 8.94	5.00 9.62 9.85	5.27 8.25 9.49	6.06 7.64 9.05	5.63 7.40 8.90
U13 _(*n* = 205)_	3.78 5.88 7.99	4.43 9.55 9.77	4.46 7.63 9.04	4.62 6.60 8.41	4.20 6.10 8.10
	*13 × 15 × 17 × 13*	*15 × 17 × 13*	*13 × 15 × 17 × 13*	*13 × 15 × 17 × 13*	*13 × 15 × 17 × 13*
	*p < 0.01*	*p < 0.01*	*p < 0.01*	*p < 0.01*	*p < 0.01*
					
	*Effect size (ES)* *(95% Confidence interval)*				
*U17 x U13*	*0.90* *(0.88–0.92)*	*0.69* *(0.67–0.71)*	*0.85* *(0.83–0.87)*	*0.94* *(0.92–0.96)*	*0.94* *(0.92–0.96)*
*U17 x U15*	*0.37* *(0.35–0.38)*	*0.34* *(0.32–0.35)*	*0.51* *(0.50–0.53)*	*0.48* *(0.47–0.50)*	*0.43* *(0.42–0.45)*
*U15 x U13*	*0.48* *(0.46–0.49)*	*0.31* *(0.29–0.32)*	*0.27* *(0.25–0.28)*	*0.39* *(0.38–0.41)*	*0.44* *(0.43–0.46)*
					
	P25 P50 P75				
Expertise	(Max. 10.0 pts)				
SC _(*n* = 414)_	5.93 8.00 9.11	9.15 9.77 9.85	6.58 8.92 9.67	6.83 8.40 9.45	6.38 8.00 9.20
RC _(*n* = 354)_	4.36 6.39 8.38	4.53 9.62 9.79	4.65 7.40 9.11	5.02 7.00 8.59	4.60 6.60 8.40
	*p < 0.01*	*p < 0.01*	*p < 0.01*	*p < 0.01*	*p < 0.01*
					
	*Effect size (ES)* *(95% Confidence interval)*				
*SC x RC*	*0.50* *(0.49–0.51)*	*0.39* *(0.38–0.41)*	*0.55* *(0.54–0.56)*	*0.57* *(0.56–0.58)*	*0.53* *(0.52–0.54)*

Factor 1: decision making; Factor 2: operational tactical principles; Factor 3: collective tactical-technical elements; Factor 4: offside rule; TCTOF-BRA Overall: tactical knowledge; P25: 25th percentile; P50: 50th percentile (median); P75: 75th percentile; Max., maximum; SC, state/national/international competitions; RC, regional competitions; U, under; ES, effect size (Cohen’s *d*). Mann–Whitney U for comparison of categories in pairs and for comparison between expertise. 13 × 15 × 17 × 13 indicates that there were significant differences between categories U13 and U15, U15 and U17, and U17 and U13.

The composite reliability of the TCTOF-BRA (tactical knowledge) was 0.89. When analyzed by factor, the values were 0.69 (Factor 1), 0.79 (Factor 2), 0.67 (Factor 3), and 0.39 (Factor 4). The overall index of internal consistency of the TCTOF-BRA (tactical knowledge) by the KR20 was 0.74. The values by dimension were 0.60, 0.67, 0.48, and 0.35, respectively. The reasons for the reduction in these values when analyzed by factor are presented in the discussion.

The ICC showed almost perfect agreement for the overall result of the TCTOF-BRA (Table 5). When analyzed by dimension, factor 1 showed almost perfect agreement and factors 2, 3, and 4 substantial agreement. The agreement results per item verified by kappa are presented in Supplementary Table 2 (Data Sheet 1) and the values of the questions that were included in the TCTOF-BRA ranged from 0.24 (fair agreement) to 0.66 (substantial agreement).

**Table 5:** Repeatability: test–retest reliability analysis.

	TCTOF-BRA (*n* = 85)			
	ICC_relative_	CI 95%	ICC_absolute_	CI 95%
Factor 1	0.86	0.78–0.91	0.85	0.77–0.90
Factor 2	0.67	0.50–0.79	0.66	0.48–0.78
Factor 3	0.61	0.40–0.75	0.61	0.40–0.75
Factor 4	0.80	0.70–0.87	0.78	0.65–0.86
TCTOF-BRA overall	0.85	0.78–0.91	0.85	0.77–0.90

Factor 1: decision making; Factor 2: operational tactical principles; Factor 3: collective tactical-technical elements; Factor 4: offside rule; ICC: Intraclass Correlation Coefficient; *p* ≤ 0.001. TCTOF-BRA Overall: tactical knowledge.

### Discussion

Study 2 aimed to investigate and present evidence of construct validity and reliability of the TCTOF-BRA. The sample size (*n* = 768) was in accordance with various types of guidance and guidelines (Guadagnoli and Velicer, 1988; Fabrigar and Wegener, 2012, p. 26; Pasquali, 2010, p. 185; Pituch and Stevens, 2016, p. 347), which allowed robust statistical analyses.

Face validity was reaffirmed in the target sample. The American Educational Research Association [AERA] et al. (2014) and Padilla and Benítez (2014) highlight the importance of analyzing, through interviews and focus groups, whether data collection procedures are adequate, how participants understand and answer items/questions (“evidence based on response processes”), and whether the test or any of its items may cause possible discomfort to the participants (evidence based on “consequences of testing”). This type of validity has received less attention and may impact other validity evidence; therefore, its application is suggested from the pilot study in investigations related to instrument validation.

Because factor analysis was not performed in the original version of the TCTOF (Serra-Olivares and García-López, 2016), in the Brazilian sample (TCTOF-BRA) it was decided to submit the instrument to EFA and CFA to determine the construct validity, which refers to the degree to which the scores of a test measure a hypothetical construct. For this, the literature suggests a “previous analysis of the items,” seeking to verify which of them are the most adequate to be included and tested in the model (Field, 2009, p. 572; Thomas et al., 2015, p. 395).

The theoretical and empirical analysis of the items showed 16 questions that met all the criteria. However, the question about “operational tactical principles: keeping/maintaining the ball possession,” was included as it presented good results and is a conceptually important item for understanding the instrument’s logic. At the same time, two questions (“the shoot works to” and “the pass works to”) were excluded because they saturate in different factors in a first EFA and because of the agreement of the main author of the instrument on the possibility of excluding them, since they have a pattern of responses that is different from the others. Thus, the Brazilian version of the TCTOF contains 15 questions (see in the Supplementary Material – Data Sheet 2 – TCTOF-BRA). The estimated time to answer these questions is approximately 10 min.

As for the cutoff points adopted, references from several areas were used, such as psychometrics, statistics, physical education, and sports. The average difficulty index of the 15 questions was 0.70, which is within criteria that are even more conservative (0.30–0.70; Nakano et al., 2015, p. 99). Item discrimination using the D index showed an average difference of 40.8 percentage points between the results of the best 27% and the worst 27%. According to Thomas et al. (2015), researchers generally seek indices equal to or greater than 0.20, which correspond to 20 percentage points. Still on discrimination, the average item-total correlation coefficient (biserial point) was 0.39. Pasquali (2018, p. 136) states that a “correlation of 0.33 is sufficiently high to indicate reasonable discrimination.” For Field (2009, p. 598), if the sample is large, even coefficients smaller than 0.30 are acceptable. Regarding the unidimensionality assumption, the average factor loading of the 15 items went from 0.21 after forced extraction of 1 factor, to 0.62 with the items saturated in the four extracted factors, that is, there was an increase of approximately 200% in the mean value of the factor loading, showing that the test should not be characterized as unidimensional (Pasquali, 2018, p. 117), as suggested by the unidimensional congruence (UniCo) and explained common variance (ECV) indicators.

Pasquali (2018, p. 116) indicates that for “a factor loading to be high, it needs to be at least 0.30.” Field (2009, p. 569) also presents 0.30 as a cutoff point, but suggests that depending on the sample size the factor loading can be significant with smaller values, for example, for a “sample of 600 participants a load >0.21 would already be significant” (Stevens, 1992, pp. 382–384). Guadagnoli and Velicer (1988) also indicated the relation of the factor loading with the sample size (0.40 and *n* > 500) and that the factor loading is affected by the type of answer, that is, in true-false type tests a load of 0.40 is enough, while for tests with answers in the Likert-type format, factor loadings in the range of 0.60 are expected for a good fit of the model. In the present study, all 15 questions had a factor loading ≥0.25. In addition, Hair et al. (2014, p. 115) indicate that for a factor loading to be significant (*p* < 0.05 and 80% power) the sample size must be ≥350. Finally, the reliability of the items by the test–retest to reach the 15 TCTOF-BRA questions was also taken into consideration. The mean value of the kappa coefficient was 0.41, considered moderate (Landis and Koch, 1977) and sufficient (Pestana and Gageiro, 2005, p. 162).

The EFA showed a model composed of four factors, with a KMO considered acceptable (medium to good) and a highly significant Bartlett test, indicating that the factor analysis is adequate. In the first factor, all the questions about “decision making” in game situations through figures were saturated; in the second factor the questions about “operational tactical principles”; in the third factor all the “collective tactical-technical elements”; and in the fourth factor, the two questions related to the “offside rule.” The total explained variance was 58.2% and the determinant did not suggest multicollinearity (highly correlated variables). According to the reference values presented by Marôco (2010, p. 51), all indices investigated in the EFA had results considered very good. Afterward, we proceeded with the CFA of the TCTOF-BRA.

Multivariate normality was not confirmed and when excluding possible outliers with *p* < 0.05, new “extreme” values emerged, as expected (Marôco, 2010, p. 143). Therefore, the model was tested in the CFA with all 768 investigated subjects. According to Marôco (2010, p. 143), high values in the items, which means they can be considered as outliers, “may reflect real observations that are necessary to remain in the analysis.” Considering that the presence of extreme values not resulting from a natural situation “may compromise the goodness of fit of the model” (Marôco, 2010, p. 64), and, therefore, if there are outliers, the model will have a bad fit, it was chosen to assume that the possible outlier cases (lower values and higher values) reflected the real tactical knowledge of these participants and, therefore, would not be initially excluded to test the model. It is worth remembering that some cases have already been discarded, according to the exclusion criteria for the final composition of the sample (796–28 = 768).

There are several estimation methods for CFA suggested in the literature for categorical variables, as is the case in the present study. Olsson et al. (2000) suggest testing different methods, as they are affected by a series of parameters, such as data distribution and sample size. Likewise, several indices have been proposed to assess the quality of the model and studies that “involve structural equation analysis do not report all these indices presented in the literature” (Marôco, 2010, p. 50). In other words, there is not only a single way to determine the validity of an instrument, but important evidence to be tested according to the latent variable/construct. Due to this scenario, two estimation methods were used, asymptotically Distribution-Free (ADF) as standard and Robust Diagonally Weighted Least Squares (RDWLS) for comparison purposes. Absolute, relative, parsimony, discrepancy, and information theory-based indices were investigated to analyze the goodness of fit of the model.

Confirmatory factor analysis by the ADF method demonstrated, from the original model (Model 1), an acceptable to very good goodness of fit (*X*^2^/df good; GFI very good; CFI very good; TLI acceptable; PGFI good; PCFI good, RMSEA very good, and ECVI very good). After the correlation between the errors of the questions “width” and “numeric superiority,” the goodness of fit was significantly (*p* < 0.001) better (*X*^2^/df closer to 1 = very good and TLI = good) and remained like this after the second-order CFA. Low ECVI values indicate external validity, that is, study population validity, in addition to that verified in the present sample. A trajectory was added only between the errors of the variables “width” and “numeric superiority,” since the modification index was greater than 11. From a theoretical point of view, this is most likely due to the interaction between these elements in the execution of the fundamental tactical principle “width and depth,” since it seeks “movements to expand the playing space that provide numerical superiority during the attack” (Costa et al., 2011, p. 516). There was no “correlation between residuals and latent factors,” which confirms the theoretical referential (Marôco, 2010, p. 183), that is, the items saturated in the four dimensions according to the theoretical basis of the study.

Complementarily, the RDWLS method confirmed the findings and showed even better quality indices, such as *X*^2^ with *p* value >0.05, *X*^2^/df < 1 and all other indices considered very good. The findings by both methods strongly indicate that we are facing a plausible/real model (Olsson et al., 2000). It is worth noting that when the model is correctly specified, different estimation methods will produce convergent results (Browne, 1984; Olsson et al., 2000), especially in large samples.

Pasquali (2010, p. 190) reports that in addition to factor analysis, construct validity can be determined using “age as a criterion when the test measures traits that are intrinsically dependent on changes in the subjects’ cognitive/affective development.” Thus, one more piece of evidence of the construct validity was investigated, using the known-groups difference method (*p* values and effect sizes), showing that young male soccer players from higher categories and who participate in state-level championships or higher have better results in the TCTOF-BRA than their peers, as expected. Several studies have even used only this method for construct validity of their tests (Mangas, 1999; Serra-Olivares and García-López, 2016; Machado and da Costa, 2020), although it is not possible to assess the internal structure (dimensionality) of the instrument, a condition that is fundamental in validation studies (American Educational Research Association [AERA] et al., 2014) and performed in the present paper.

Regarding reliability of the TCTOF-BRA, the internal consistency, a measure that represents the congruence that each item of the instrument has with the rest of the items, was investigated by the composite reliability (CR) and by the Kuder–Richardson coefficient (KR20) for dichotomous variables; and temporal stability, referring to consistency of the test scores over time, by the test–retest reliability using the intraclass correlation coefficient (ICC).

The TCTOF-BRA showed substantial internal consistency, either by composite reliability (0.89) or by the KR20 coefficient (0.74), as the values were above 0.60 (Landis and Koch, 1977; Bagozzi and Yi, 1988) and in a more conservative situation above 0.70 (Hair et al., 2014, p. 123).

When the internal consistency was analyzed by factor, one of them was observed below 0.60 in CR (Factor 4). Valentini and Damásio (2016) bring a reflection on the use of composite reliability (CR) as a precision indicator. According to the authors, single and fixed cutoff points for CR do not seem justifiable, as CR results vary in function of factor loadings and number of items. For example: Factor 4 with two items and factor loadings of 0.44 and 0.42 showed a CR of 0.39; if this same factor had 10 items, half of them with a factor loading of 0.44 and half with 0.42, preserving what was found, the composite reliability would change to 0.76. In the KR20 coefficient, when the analysis is by factor, the consistency was from fair (Factor 4), passing through moderate (Factors 1 and 3), to substantial consistency (Factor 2). Maroco et al. (2008, p. 646), when analyzing the internal consistency by Cronbach’s alpha, similar to KR20, confirm that the lower values found when the analysis is by factor, “is due more to the reduced number of items than to the loss of measure consistency.” Therefore, caution must be taken when analyzing these values individually and by factor/dimension.

The temporal stability of the TCTOF-BRA was verified by the test–retest in 11.1% of the sample, between 7 and 17 days after the first application of the test. Studies have used at least 10% of the target sample (Greco et al., 2015; Machado and da Costa, 2020) and the literature suggests an interval long enough to allow participants to forget about the test, since what it is intended to assess is the ability and not the capacity to remember the answers; and short enough so that “changes in maturation, development and learning” do not occur, for example, that change the test results (Thomas et al., 2015, p. 375; Zanon and Filho, 2015, pp. 89–90). In this study, we chose to present the ICC in an absolute and relative way, the first being used to “verify if the results are the same” and the second “if the results are similar” (Pestana and Gageiro, 2005, p. 526).

The study showed an almost perfect agreement of the TCTOF-BRA scores over time (0.85). By factor, stability went from substantial to almost perfect (0.61–0.86). Validation studies in the area have also adopted the cutoff points of Landis and Koch (1977) to assess the intraclass correlation coefficient in test–retest situations, as in Costa et al. (2017), or very similar to them, as in the case of Greco et al. (2015) when adopting Szklo and Nieto (2000): 0.40–0.74 satisfactory agreement and ≥ 0.75 excellent agreement. Finally, the reliability of this study followed the guidance of Hair et al. (2014, p. 123) with the assessment of internal consistency by CR and KR20 and stability by the test–retest, using an intraclass correlation coefficient.

## General Discussion

The main hypothesis of the study was that the TCTOF is an instrument capable of evaluating also the tactical knowledge of young male soccer players in the Brazilian context. After translation, adaptation, content and face validity, item analysis, construct validity, and reliability determination, the results allow confirmation of the hypothesis that the TCTOF-BRA is an instrument with valid and reliable measures for use in young male Brazilian soccer players.

Through the aforementioned procedures, modifications were made and deserve to be registered. The original Spanish version of the *Test de Conocimiento Táctico Ofensivo en Fútbol* has 52 questions. In the first part, there are questions that represent knowledge about: roles and positions, offside rule, individual technical-tactical elements, operational tactical principles (OTP), relationship between individual technical-tactical elements and the OTP, and collective technical-tactical elements. In the second part, there are game situations that represent knowledge about: decision making in situations of keeping/maintaining the ball, decision making in situations of advancing/progressing, and decision making in situations of attacking/trying to score the goal and the offside rule. In summary, of the 52 questions, 51 underwent cross-cultural adaptation and content validation, with satisfactory results in 43 (84.3%). Of the questions applied in the target sample, 16 met all the established criteria, two were excluded, and one was included later.

Therefore, the Portuguese (Brazilian population) version of the TCTOF, named the *Teste de Conhecimento Tático Ofensivo no Futebol (TCTOF-BRA)*, contains 15 questions (Supplementary Material – Data Sheet 2). The EFA and CFA showed a very good model goodness of fit composed of a second-order factor (tactical knowledge) and four first-order factors. Factor 1 included all six figure-type questions about “decision making” in situations of keeping/maintaining, advancing/progressing, and attacking/trying to score the goal; factor 2 included the three questions about “OTP”; factor 3, four questions about “collective tactical-technical elements”; and factor 4, two questions related to the “offside rule.” Questions related to roles and positions, individual technical-tactical elements, and the relationship of individual technical-tactical elements with the OTP were not relevant to determine the tactical knowledge of young male Brazilian players.

Thus, based on the literature and the findings of the present study, it is possible to denominate the TCTOF-BRA as a test that: (a) assesses the tactical domain from an expanded and cognitive perspective (Abernethy et al., 1993; McPherson, 1994; Rechenchosky et al., 2021); (b) is in accordance with cognitive abilities theory of Anderson (1982) and the structure of knowledge by McPherson and Thomas (1989); (c) predominantly uses declarative/explicit/cognitive memory (Michaelian, 2010), since “more than one system can contribute to performance in a particular task” and “the operations of many forms of memory, including cognitive memory, are sometimes expressed implicitly rather than explicitly” (Schacter and Tulving, 1994, pp. 12–31); (d) is based on interactionism theories and presents evidence of ecological validity, since by having operational tactical principles as a theoretical basis, it allows similarities between the test and what happens in matches; and (e) assesses tactical knowledge (overall result), based on specific knowledge (Factors 2, 3, and 4) and decision making (Factor 1). It is worth highlighting that the TCTOF-BRA “decision making” construct is in agreement with the structure of knowledge presented by McPherson and Thomas (1989) and McPherson (1994), since in game situations represented by pictures the participant is requested to declare “what to do and how to do it” (concepts of action) and in an undeclared way analyze “why do it” (concept of condition) and “for what reason are you doing it” (concept of objective).

Therefore, the TCTOF-BRA assesses tactical knowledge based on specific knowledge (operational tactical principles, collective tactical-technical elements, and rules) and decision making (“what to do,” “why do it,” “how to do it,” and “for what reason are you doing it”). For part of the literature (Anderson, 1982; Abernethy et al., 1993; McPherson, 1994), this specific knowledge refers to declarative knowledge, as it involves factual knowledge about the modality, and decision making refers to procedural knowledge, because it allows the use of this knowledge in situations like “if = then.”

Although in criterion-related validity, which represents the degree to which the test scores are related to some recognized standard or criterion (Thomas et al., 2015, p. 360), scores given by the coaches can be considered as measures to be correlated with the test results, there is an important limitation in this method when involving a large number of judges/coaches, as was the case in the present study. It is not possible to guarantee the inter-rater reliability of the coaches regarding the scores given for the tactical knowledge of their players, which are further analyzed together with the scores given by other coaches. Therefore, and corroborating Thomas and Thomas (1994) regarding the classification of athletes as experts by different researchers, this type of validity evidence did not advance in the study because we did not consider the scores of different coaches, without any evidence of inter-rater reliability, as reference standard. This may justify the infrequent use of this type of validity (20.7%), as seen in the review by Rechenchosky et al. (2021).

Finally, tests developed or validated with a Brazilian sample to assess the tactical knowledge or behavior of young male soccer players are rare. The first of these is the TCTP-OE (Greco et al., 2015), which assesses tactical-technical behavior, and is theoretically based on general tactical principles and meets eight of the 13 quality criteria proposed by Rechenchosky et al. (2021). The second was the TacticUP (Machado and da Costa, 2020), which assesses tactical knowledge, and is theoretically based on the core tactical principles of soccer and meets five quality criteria. Finally, the TCTOF-BRA is a test that assesses tactical knowledge, and is theoretically based on operational tactical principles and meets 11 criteria and has shown satisfactory psychometric properties, and may be used by professors, coaches, and researchers to identify potential talents in sport, to classify and compose teams in training sessions, as suggested by Praça et al. (2017), to guide the teaching-learning-training process, and contribute to monitoring the tactical dimension of young soccer players in the Brazilian context. These assumptions tend to be confirmed with evidences of convergent validity (tactical knowledge × tactical behavior).

## Highlights, Limitations, and Future Directions

According to the *Standards* (American Educational Research Association [AERA] et al., 2014, p. 13) and Urbina (2014) on what is expected in a validation process, this study furnishes relevant evidence, for the user to evaluate “the evidence in the particular setting in which the test is to be used.” Some highlights are presented.

The study involved a relatively large sample, the largest among the tests that assess the tactical knowledge or tactical behavior of young soccer players (Rechenchosky et al., 2021). Although there was no stratification for the selection of clubs, the sample consisted of players from 24 states and from all five regions of Brazil, who train in a systematic way, seeking high performance and participation in competitions.

The TCTOF-BRA, which is a questionnaire that assesses tactical knowledge and not tactical behavior/performance, has a well-defined theoretical basis (operational tactical principles), contributing to the ecological validity, and has equations in which the results are calculated based on the characteristics of each question, that is, the properties of each item regarding the difficulty, discrimination, and unidimensionality are preserved to obtain the scores of “tactical knowledge” and of the dimensions “decision making,” “operational tactical principles,” “collective tactical-technical elements,” and “rules.” The final model was very parsimonious, that is, it was practically not artificially improved, since the goodness of fit was considered very good from the first model, which indicates that it is something real. In addition, the TCTOF-BRA is one of the tests that assess the tactical dimension and which includes a greater number of investigated and reported psychometric properties, advancing from 9 (TCTOF Spanish version) to 11 quality criteria, of the 13 recently proposed by Rechenchosky et al. (2021, p. 2053), being: sample description, sample size, sample heterogeneity, content validity, face validity, construct validity, internal consistency, repeatability/intrarater reliability, EFA, CFA, and V/R (validity and reliability).

Although the CFA indicates external validity of the TCTOF-BRA, that is, that the test is valid for the study population, and even involving players from 24 Brazilian states, it is proposed that future studies use Brazilian samples with different characteristics and that are also composed of school students so that normative values can be established. It is also suggested that the validity be investigated based on relationships with external measures (American Educational Research Association [AERA] et al., 2014), through concurrent validity (same construct) and convergent validity (tactical knowledge × tactical behavior), as well as the neural mechanisms involved in the tasks of the test.

## Conclusion

The *Teste de Conhecimento Tático Ofensivo no Futebol (TCTOF-BRA)*, Brazilian version with 15 questions from the *Test de Conocimiento Táctico Ofensivo en Fútbol*, presented satisfactory evidence regarding cross-cultural adaptation, content validity, face validity, construct validity, internal consistency, and temporal stability, proving to be a test with valid and reliable measures for the assessment of tactical knowledge (declarative and theoretical procedural), based on specific knowledge and decision making (cognitive domain), of young male Brazilian soccer players from 12 to 17.9 years old.

## Data Availability Statement

The original contributions presented in the study are included in the article/**Supplementary Material**; further inquiries can be directed to the corresponding author.

## Ethics Statement

The studies involving human participants were reviewed and approved by Ethics Committee of State University of Maringá (CAAE 08918619.3.0000.0104; Opinion 3.208.874). Written informed consent to participate in this study was provided by the participants’ legal guardian/next of kin.

## Author Contributions

LR, PB, JS-O, and WR designed the study (conceptualization). LR, VM, and MJ planned and conducted the data collection (methodology and investigation). LR performed the data analysis (formal analysis). LR, VM, MJ, PB, JS-O, and WR participated in writing and reviewing the manuscript. All authors contributed to the article and approved the submitted version.

## Funding

This work was written with the support of the Programa Academia & Futebol, of the Secretaria Nacional de Futebol e Defesa dos Direitos do Torcedor/Secretaria Especial do Esporte/Ministério da Cidadania (SNFDT/SEESP/MC) Brazilian Government. This study was financed in part by the Coordenação de Aperfeiçoamento de Pessoal de Nível Superior – Brasil (CAPES) – Finance Code 001, and the Programa de Pós-Graduação Associado em Educação Física (PEF-UEM/UEL), through payment of the publication fee.

## Conflict of Interest

The authors declare that the research was conducted in the absence of any commercial or financial relationships that could be construed as a potential conflict of interest.

## Publisher’s Note

All claims expressed in this article are solely those of the authors and do not necessarily represent those of their affiliated organizations, or those of the publisher, the editors and the reviewers. Any product that may be evaluated in this article, or claim that may be made by its manufacturer, is not guaranteed or endorsed by the publisher.

## Figures and Tables

**Table:** 

ADF	Asymptotically distribution-free
CCV	Coefficient of content validity
CCV_i_	Coefficient of content validity by item/question and criterion
CCV_t_	Coefficient of content validity total of the instrument, total per equivalence/parameter or total per question
CFA	Confirmatory factor analysis
CFI	Comparative fit index
CIs	Confidence intervals
CR	Composite reliability
df	Degrees of freedom
ECV	Explained common variance
ECVI	Expected cross-validation index
EFA	Exploratory factor analysis
ES	Effect size
GFI	Goodness of fit index
ICC	Intraclass correlation coefficient
KMO	Kaiser–Meyer–Olkin
KR20	Kuder–Richardson coefficient
OPT	Operational tactical principles
PCFI	Parsimony CFI
PGFI	Parsimony GFI
RMSEA	Root mean square error of approximation
RDWLS	Robust diagonally weighted least squares
SD	Standard deviation
*se*	Asymptotic standard error
TCTOF	*Test de Conocimiento Táctico Ofensivo en Fútbol*
TCTOF-BRA	*Teste de Conhecimento Tático Ofensivo no Futebol*
TLI	Tucker Lewis fit index
UniCo	Unidimensional congruence
X^2^	Chi-square
