# Supplementary material for: Adaptation and Validation of a Test for the Evaluation of Tactical Knowledge in Soccer: Test de Conocimiento Táctico Ofensivo en Fútbol for the Brazilian Context (TCTOF-BRA)
Source: Front Psychol. 2022 Jul 14;13:849255. doi: 10.3389/fpsyg.2022.849255 (PMC9330055; doi:10.3389/fpsyg.2022.849255)
Supplement: Supplementary file 1 [file Data_Sheet_1.pdf]

Supplementary Material

Table 1. Empirical analysis of the items (Study 1).

Table 2. Theoretical (Study 1) and empirical item analysis (Study 2).

Frame 1. Indices and reference values for goodness of fit analysis.

Frame 2. Equations to estimate tactical knowledge and factors 1, 2, 3, and 4.

Figure 1. First order confirmatory factor analysis (Model 1).

Figure 2. Confirmatory factor analysis after error correlation (Model 2).

Table 1. Empirical analysis of the items (Study 1).

Questions	Difficulty	Discrimination	
TCTOF – Part 1	DI	D	R_{pb}
**1) What role CANNOT adopt an attacking player?**	**0.68**	**44**	**0.36**
2) What position CANNOT have a soccer player?	0.92	17	0.43
3) What position CANNOT have a defender?	0.95	13	0.58
**4) What position CANNOT have a midfielder?**	**0.90**	**29**	**0.50**
5) What position CANNOT have a forward?	0.93	24	0.39
**6) A soccer player is offside position when:**	**0.74**	**44**	**0.33**
7) A shoot is:	0.97	8	0.38
8) Screening or shielding the ball is:	0.95	16	0.31
9) Running with the ball is:	0.96	13	0.38
**10) A control is:**	**0.85**	**32**	**0.40**
11) A faint is:	0.34	-18	-0.05
12) A pass is:	1.00	0	0
13) A dribble is:	0.78	21	0.23
14) A check is:	0.72	16	0.15
**15) A check to is:**	**0.64**	**42**	**0.33**
**16) A check away is:**	**0.51**	**58**	**0.46**
17) What do you understand about keeping possession of the ball?	0.92	20	0.25
**18) What do you understand about moving towards the opponent's goal?**	**0.62**	**36**	**0.33**
19) What do you understand about attacking the opponent's goal?	0.90	12	0.20
20) Dribbling is useful to:	0.51	36	0.27
21) The shoot works to:	0.77	24	0.20
22) Checking away from a defender is useful to:	0.31	12	0.16
23) Controlling the ball is useful to:	0.28	12	0.14
24) The pass works to:	0.32	32	0.25
25) Running with the ball is useful to:	0.31	20	0.20
**26) Shielding/screening is useful to:**	**0.81**	**32**	**0.37**
**27) Give-and-go or wall pass is:**	**0.65**	**44**	**0.43**
**28) Providing "width" to the game is:**	**0.63**	**66**	**0.58**
**29) A "triangulation" is:**	**0.86**	**28**	**0.35**
**30) Providing "depth" to the attack is:**	**0.49**	**60**	**0.44**
**31) Numeric superiority situations in attack are:**	**0.72**	**34**	**0.33**
**32) Creating free spaces is:**	**0.77**	**40**	**0.44**
**33) A "permute" in attack is:**	**0.70**	**26**	**0.38**
**34) An overlapping is:**	**0.72**	**42**	**0.35**
**35) Temporize the game when attacking is:**	**0.86**	**32**	**0.38**
TCTOF – Part 2			
1. Situation of progressing/moving towards the opponent's goal	0.09	-16	-0.11
**2. Situation of attacking the goal/trying to score a goal**	**0.88**	**24**	**0.32**
3. Situation of keeping/maintaining possession of the ball	0.49	28	0.28
**4. Situation of progressing/moving towards the opponent's goal**	**0.82**	**40**	**0.45**

**5. Situation of keeping/maintaining possession of the ball**	**0.81**	**40**	**0.47**
**6. Situation of keeping/maintaining possession of the ball**	**0.36**	**52**	**0.36**
**7. Situation of progressing/moving towards the opponent's goal**	**0.59**	**36**	**0.37**
8. Situation of attacking the goal/trying to score a goal	0.73	4	0.07
9. Situation of keeping/maintaining possession of the ball	0.15	12	0.16
**10. Situation of progressing/moving towards the opponent's goal**	**0.65**	**48**	**0.37**
11. Situation of keeping/maintaining possession of the ball	0.59	28	0.19
**12. Situation of progressing/moving towards the opponent's goal**	**0.75**	**48**	**0.42**
13. Situation of keeping/maintaining possession of the ball	0.23	12	0.10
14. Situation of progressing/moving towards the opponent's goal	0.29	20	0.14
**15. Situation of progressing/moving towards the opponent's goal**	**0.46**	**55**	**0.40**
**16. Situation about the offside rule**	**0.74**	**38**	**0.48**

DI: difficulty index; D: discrimination index (method 27); R_{pb}: item-total point-biserial correlation. Questions in bold = DI ≥ 0.10 and ≤ 0.90, D ≥ 20 (0.20), and R_{pb}: ≥ 0.30.

Table 2. Theoretical (Study 1) and empirical item analysis (Study 2).

Questions	Cross-cultural adaptation	Content Validity	NQ	Difficulty	Discrimination		U	A	
**TCTOF – Part 1**	**CCV_t**	**CCV_i**	**%**	**DI**	**D**	**R_{pb}**	**Factor loading**	**Kappa**	***p***
1) What role CANNOT adopt an attacking player?	0.96	PR TR	10.8	**0.73**	**36.5**	**0.32**	**0.30**	**0.42**	<0.01
2) What position CANNOT have a soccer player?	0.90	CL TR	0.8	0.98	6.3	0.19	0.24	-	-
3) What position CANNOT have a defender?	0.94	CL TR	0.1	0.97	9.6	0.29	**0.39**	**1.00**	<0.01
4) What position CANNOT have a midfielder?	0.90	CL TR	0.1	0.98	6.7	0.21	0.28	-0.01	*0.91*
5) What role CAN'T adopt an attacking player?	0.90	CL TR	0.4	0.98	6.3	0.24	**0.31**	-0.01	*0.91*
6) A soccer player is offside position when:	0.86	CL PR TR	1.3	**0.83**	**31.7**	**0.36**	**0.39**	**0.44**	<0.01
7) A shoot is:	0.91	CL PR TR	-	0.99	3.8	0.19	0.25	-	-
8) Screening or shielding the ball is:	0.96	CL PR TR	1.7	0.94	6.7	0.12	0.11	**0.37**	<0.01
9) Running with the ball is:	0.96	CL PR TR	0.3	0.97	7.7	0.23	0.27	-	-
10) A control is:	0.92	CL PR TR	0.3	0.97	5.8	0.23	0.13	**0.65**	<0.01
11) A faint is:	1.00	CL PR TR	0.8	**0.43**	**37.0**	0.26	0.20	**0.26**	<0.01
12) A pass is:	0.99	CL PR TR	-	1.00	0.5	0.06	0.06	-	-
13) A dribble is:	0.95	CL PR TR	0.8	**0.77**	**24.2**	0.22	0.17	**0.42**	<0.01
14) A check is:	0.95	CL PR TR	2.0	**0.79**	17.4	0.19	0.16	**0.26**	<0.01
15) A check to is:	0.95	CL PR TR	6.1	**0.60**	**36.1**	0.29	0.24	**0.23**	<0.05
16) A check away is:	0.96	CL PR TR	10.4	**0.43**	**32.6**	0.26	0.20	**0.25**	<0.05
17) What do you understand about keeping possession of the ball?	1.00	CL PR TR	-	0.95	14.4	0.27	**0.32**	**0.66**	<0.01
18) What do you understand about moving towards the opponent's goal?	0.96	CL PR TR	0.1	**0.79**	**46.2**	**0.46**	**0.50**	**0.52**	<0.01
19) What do you understand about attacking the opponent's goal?	1.00	CL PR TR	1.4	**0.75**	**48.1**	**0.46**	**0.50**	**0.28**	<0.01
20) Dribbling is useful to:	0.95	CL PR TR	0.3	**0.15**	9.6	0.10	0.01	**0.23**	<0.01
21) The shoot works to:	0.97	CL PR TR	0.4	**0.64**	**48.6**	**0.39**	**0.40**	**0.44**	<0.01
22) Checking away from a defender is useful to:	0.99	CL PR TR	2.3	**0.26**	**35.8**	**0.31**	0.27	**0.21**	<0.05
23) Controlling the ball is useful to:	0.99	CL PR	0.3	**0.28**	15.6	0.15	0.07	**0.30**	<0.01
24) The pass works to:	0.96	CL PR TR	0.1	**0.41**	**40.9**	**0.34**	**0.31**	**0.24**	<0.01
25) Running with the ball is useful to:	0.96	CL PR TR	0.3	**0.29**	**26.4**	0.21	0.13	**0.22**	<0.05
26) Shielding/screening is useful to:	0.98	CL PR TR	0.7	**0.65**	**33.2**	0.29	0.26	**0.38**	<0.01
27) Give-and-go or wall pass is:	0.98		3.5	**0.68**	**27.9**	0.26	0.25	**0.29**	<0.01

28) Providing “width” to the game is:	0.95	CL PR TR	0.8	**0.78**	**35.3**	**0.39**	**0.41**	**0.24**	<0.01
29) A “triangulation” is:	0.96	CL PR TR	0.8	**0.84**	**26.0**	**0.32**	**0.34**	0.11	*0.29*
30) Providing “depth” to the attack is:	0.96	CL PR TR	0.9	**0.61**	**40.8**	**0.35**	**0.33**	**0.27**	<0.01
31) Numeric superiority situations in attack are:	0.96	CL PR TR	1.0	**0.76**	**37.7**	**0.38**	**0.41**	**0.46**	<0.01
32) Creating free spaces is:	0.99	CL PR TR	0.4	**0.75**	**35.6**	**0.34**	**0.35**	**0.27**	<0.01
33) A “permute” in attack is:	0.80	PR TR	13.8	**0.75**	**33.1**	**0.31**	0.29	**0.53**	<0.01
34) An overlapping is:	0.85	CL PR TR	0.9	**0.74**	**36.1**	**0.34**	**0.37**	0.18	*0.10*
35) Temporize the game when attacking is:	0.92	CL PR TR	1.8	**0.88**	18.9	0.26	0.27	0.08	*0.44*
**TCTOF – Part 2**									
1. Situation of progressing/moving towards the opponent’s goal	0.96	CL PR TR	0.1	**0.18**	6.3	0.07	- 0.01	**0.26**	<0.01
2. Situation of attacking the goal/trying to score a goal	1.00	CL PR TR	-	**0.78**	**54.8**	**0.51**	**0.58**	**0.40**	<0.01
3. Situation of keeping/maintaining possession of the ball	0.96	CL PR TR	0.1	**0.49**	**32.7**	0.29	0.24	**0.39**	<0.01
4. Situation of progressing/moving towards the opponent’s goal	0.96	CL PR TR	-	**0.82**	**36.1**	**0.40**	**0.39**	**0.49**	<0.01
5. Situation of keeping/maintaining possession of the ball	1.00	CL PR TR	-	**0.85**	**26.0**	**0.32**	**0.34**	0.20	*0.07*
6. Situation of keeping/maintaining possession of the ball	1.00	CL PR TR	0.5	**0.37**	**39.9**	**0.32**	**0.31**	**0.51**	<0.01
7. Situation of progressing/moving towards the opponent’s goal	1.00	CL PR TR	-	**0.63**	**26.9**	0.26	0.24	**0.37**	<0.01
8. Situation of attacking the goal/trying to score a goal	1.00	CL PR TR	-	**0.65**	**25.0**	0.23	0.24	**0.34**	<0.01
9. Situation of keeping/maintaining possession of the ball	1.00	CL PR TR	-	**0.22**	19.7	0.15	0.11	**0.25**	<0.05
10. Situation of progressing/moving towards the opponent’s goal	1.00	CL PR TR	0.1	**0.61**	**62.5**	**0.49**	**0.53**	**0.57**	<0.01
11. Situation of keeping/maintaining possession of the ball	1.00	CL PR TR	0.3	**0.68**	**34.6**	**0.30**	0.29	**0.31**	<0.01
12. Situation of progressing/moving towards the opponent’s goal	1.00	CL PR TR	0.1	**0.59**	**53.8**	**0.46**	**0.48**	**0.24**	<0.05
13. Situation of keeping/maintaining possession of the ball	0.96	CL PR TR	-	**0.25**	15.4	0.14	0.08	**0.26**	<0.05
14. Situation of progressing/moving towards the opponent’s goal	1.00	CL PR TR	0.1	**0.26**	**20.2**	0.18	0.16	0.19	*0.08*
15. Situation of progressing/moving towards the opponent’s goal	0.96	CL PR TR	0.5	**0.38**	**37.2**	**0.31**	**0.30**	**0.45**	<0.01
16. Situation about the offside rule	1.00	CL PR TR	-	**0.74**	**38.0**	**0.35**	**0.38**	**0.41**	<0.01

NQ: not attended questions; U: unidimensionality; A: agreement; CCV_t: coefficient of total content validity per question (average of equivalences); CCV_i: coefficient of content validity by item/question and criterion; DI: difficulty index; R_{pb}: item-total point-biserial correlation; CL: clarity of language; PR: practical relevance; TR: theoretical relevance; White space in any of the content validity criteria indicates a value below the threshold (0.70). Values in bold indicate satisfactory results in relation to the cutoff points adopted, as follows: ≥ 0.10 DI ≤ 0.90; D index ≥ 20 (0.20); Rpb ≥ 0.30; factor loading ≥ 0.30; kappa agreement > 0.20, and $p < 0.05$. Values that met the criteria are in bold and the 16 questions that met all the criteria are in gray.

Frame 1. Indices and reference values for goodness of fit analysis.

Statistic	**Reference values** (model adjustment)			
X^2 ; p-value	The smaller, the better; $p > 0.05$			
**Absolute indices**				
X^2/df	~ 1 Very good	< 2 Good	2 to 5 Acceptable	> 5 Bad
GFI	≥ 0.95 Very good	0.9 to 0.95 Good	0.8 to 0.9 Acceptable	< 0.8 Bad
**Relative indices**				
CFI<br>TLI	≥ 0.95 Very good	0.9 to 0.95 Good	0.8 a 0.9 Acceptable	< 0.8 Bad
**Parsimony indices**				
PGFI<br>PCFI	≥ 0.8 Very good	0.6 to 0.8 Good	< 0.6 Bad	
**Population discrepancy index**				
RMSEA	≤ 0.05 Very good	0.05 to 0.10 Good<br>$p \geq 0.05$	> 0.10 Unacceptable	
**Indices based on information theory**				
ECVI<br>MECVI	The smaller, the better			

X^2: chi-square; df: degrees of freedom; GFI: goodness of fit index; CFI: comparative fit index; TLI: Tucker-Lewis index; PGFI: parsimony goodness of fit index; PCFI: parsimony comparative fit index; RMSEA: root mean square error of approximation; ECVI: expected cross-validation index; MECVI: modified expected cross-validation index. Source: Marôco (2010, p. 51).

Frame 2. Equations to estimate tactical knowledge and factors 1, 2, 3, and 4.

Variable	Equation
**Tactical Knowledge**	$0.045xQ_1 + 0.214xQ_2 + 0.301xQ_3 + 0.337xQ_4 + 0.083xQ_5 + 0.255xQ_6 + 0.07xQ_7 + 0.315xQ_8 + 0.403xQ_9 + 0.295xQ_{10} + 0.125xQ_{11} + 0.559xQ_{12} + 0.341xQ_{13} + 0.179xQ_{14} + 0.252xQ_{15}$.
**Factor 1**	$0.006xQ_1 + 0.029xQ_2 + 0.04xQ_3 + 0.045xQ_4 + 0.011xQ_5 + 0.034xQ_6 + 0.009xQ_7 + 0.042xQ_8 + 0.104xQ_9 + 0.076xQ_{10} + 0.032xQ_{11} + 0.145xQ_{12} + 0.088xQ_{13} + 0.046xQ_{14} + 0.034xQ_{15}$.
**Factor 2**	$0.01xQ_1 + 0.046xQ_2 + 0.064xQ_3 + 0.001xQ_4 + 0.001xQ_6 + 0.001xQ_8 + 0.002xQ_9 + 0.001xQ_{10} + 0.001xQ_{11} + 0.002xQ_{12} + 0.001xQ_{13} + 0.001xQ_{14} + 0.001xQ_{15}$.
**Factor 3**	$0.003xQ_1 + 0.013xQ_2 + 0.018xQ_3 + 0.331xQ_4 + 0.082xQ_5 + 0.25xQ_6 + 0.069xQ_7 + 0.019xQ_8 + 0.025xQ_9 + 0.018xQ_{10} + 0.008xQ_{11} + 0.034xQ_{12} + 0.021xQ_{13} + 0.011xQ_{14} + 0.015xQ_{15}$.
**Factor 4**	$0.004xQ_1 + 0.021xQ_2 + 0.029xQ_3 + 0.033xQ_4 + 0.008xQ_5 + 0.025xQ_6 + 0.007xQ_7 + 0.119xQ_8 + 0.039xQ_9 + 0.029xQ_{10} + 0.012xQ_{11} + 0.055xQ_{12} + 0.033xQ_{13} + 0.017xQ_{14} + 0.095xQ_{15}$.

Factor 1 = decision making; Factor 2 = operational tactical principles; Factor 3 = collective tactical-technical elements; Factor 4 = offside rule; Q = TCTOF-BRA questions. Note: for every correct question, Q = 1; constants obtained from factor score weights calculated by AMOS.

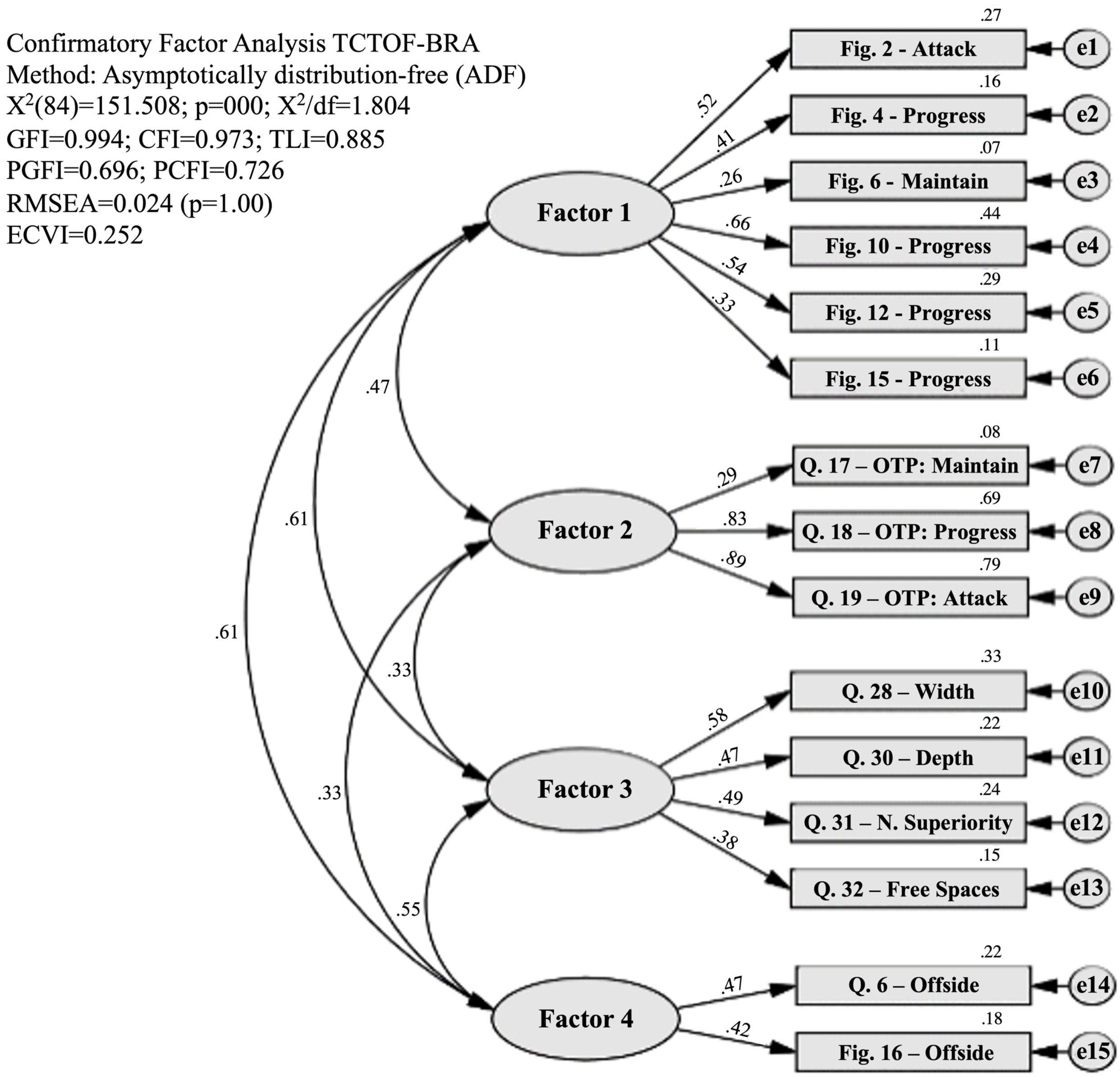

Figure 1. First order confirmatory factor analysis (Model 1). Source: the authors.

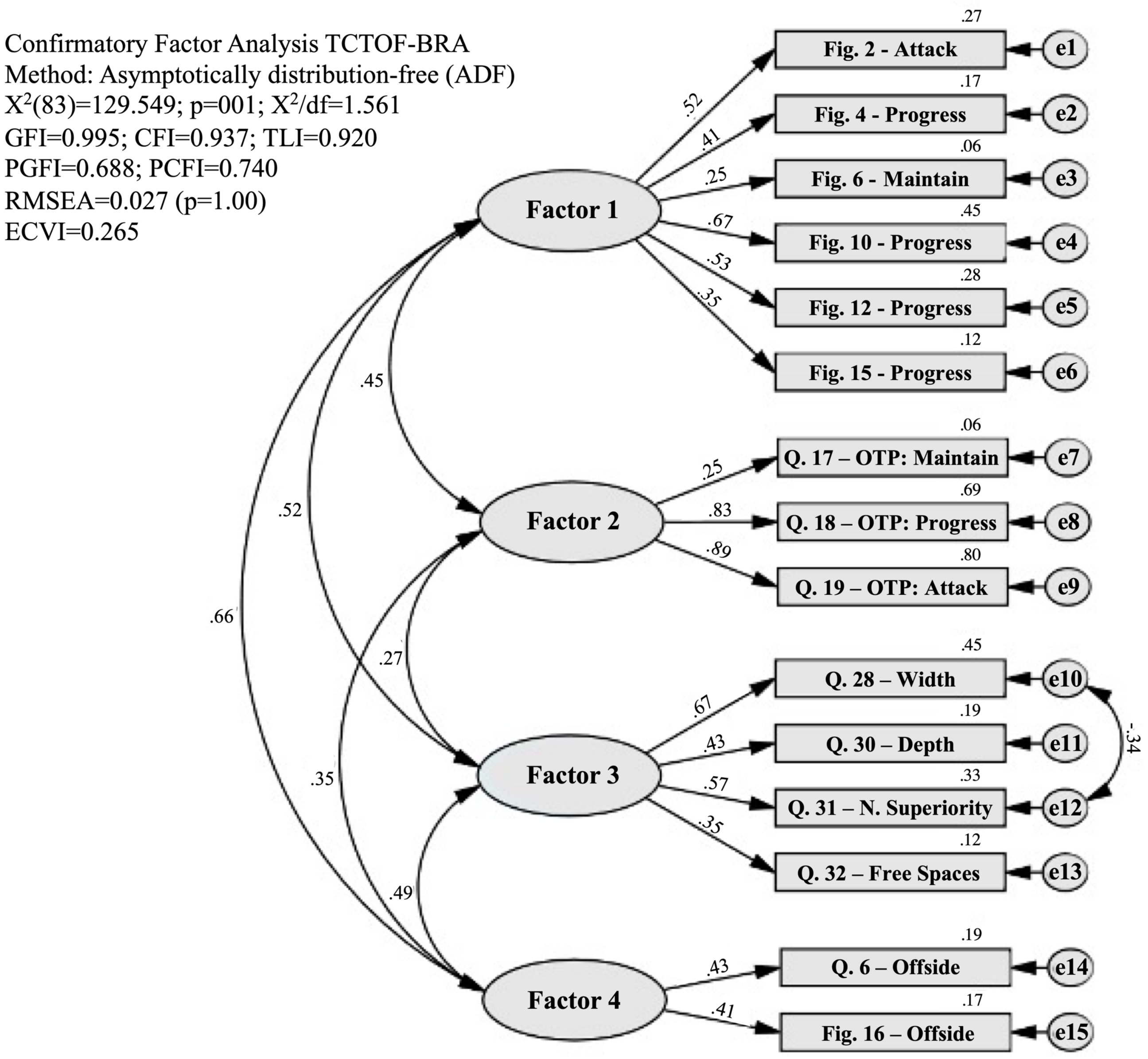

Figure 2. Confirmatory factor analysis after error correlation (Model 2). Source: the authors.
