# Supplementary material for: Adaptation and Validation of a Test for the Evaluation of Tactical Knowledge in Soccer: Test de Conocimiento Táctico Ofensivo en Fútbol for the Brazilian Context (TCTOF-BRA)
Source: Front Psychol. 2022 Jul 14;13:849255. doi: 10.3389/fpsyg.2022.849255 (PMC9330055; doi:10.3389/fpsyg.2022.849255)
Supplement: Supplementary file 2 [file Data_Sheet_2.pdf]

TESTE DE CONHECIMENTO TÁTICO OFENSIVO NO FUTEBOL

TCTOF-BRA

I) Nome completo: ____

II) Clube/instituição: ____

III) Data de nascimento: _____/_____/_______ Data da avaliação: _____/_____/_______

IV) Cidade e estado de nascimento: _____________________________________

V) Há quantos anos você treina futebol (não considerar futsal; apenas futebol)?

Menos de 1 ano ☐	3 a 4 anos ☐	6 a 7 anos ☐	9 a 10 anos ☐
1 a 2 anos ☐	4 a 5 anos ☐	7 a 8 anos ☐	Mais de 10 anos ☐
2 a 3 anos ☐	5 a 6 anos ☐	8 a 9 anos ☐	

Não participo regularmente de treinos de futebol ☐ ← *Se marcou essa opção, vá para a questão IX)*

VI) Posição de jogo que mais atua: _____________________________________

VII) Quantas vezes por semana você treina futebol? 1 ☐ 2 ☐ 3 ☐ 4 ☐ 5 ☐ 6 ☐ 7 ☐

VIII) Qual o tempo de duração de cada treino? 1h ☐ 1h15 ☐ 1h30 ☐ 2h ☐ Outro:_____

IX) Você já treinou ou treina (regularmente) outra modalidade esportiva? Não ☐ Sim ☐
Se sim, quais? ____

Parte 1

Atenção!

Da questão 1 a 8 existe apenas UMA resposta correta. Assinale com um "X" ou "O".

1) O que você entende por manter a posse de bola?

a) Conservar a posse de bola e não perdê-la.
b) Progredir em direção ao gol adversário.
c) Tentar marcar um gol.

2) O que você entende por avançar em direção ao gol adversário?

a) Conservar a posse de bola e não perdê-la.
b) Progredir em direção ao gol adversário.
c) Tentar marcar um gol.

3) O que você entende por atacar a meta adversária?

a) Conservar a posse de bola e não perdê-la.
b) Progredir em direção ao gol adversário.
c) Tentar marcar um gol.

4) Dar amplitude ao ataque é:

a) Colocar a bola em jogo mediante uma estratégia individual ou coletiva.
b) O deslocamento dos jogadores no ataque em direção ao gol adversário, ocupando razoavelmente o espaço de jogo.
c) O deslocamento dos jogadores no ataque, aumentando "até as laterais" a distância entre os mesmos e ocupando razoavelmente o espaço de jogo.

5) Dar profundidade ao ataque é:

a) Colocar a bola em jogo mediante uma estratégia individual ou coletiva.
b) O deslocamento dos jogadores no ataque em direção ao gol adversário, ocupando razoavelmente o espaço de jogo.
c) O deslocamento dos jogadores no ataque, aumentando "até as laterais" a distância entre os mesmos e ocupando razoavelmente o espaço de jogo.

6) Criar situações de superioridade numérica no ataque são:

a) Deslocamentos dos jogadores no ataque, que têm como objetivo conseguir uma situação de jogo em que haja mais atacantes que defensores.
b) Deslocamentos dos jogadores no ataque, com bola e sem bola, criando espaços livres.
c) Deslocamentos dos jogadores no ataque, que têm como objetivo conseguir uma situação de jogo em que haja mais defensores que atacantes.

7) Criar espaços livres são:

a) Deslocamentos dos jogadores no ataque, que têm como objetivo conseguir uma situação de jogo em que haja mais atacantes que defensores.
b) Deslocamentos dos jogadores no ataque, reduzindo a distância entre os mesmos e ocupando razoavelmente o espaço de jogo.
c) Deslocamentos no ataque que provocam a saída dos defensores da zona que ocupam, com a finalidade de facilitar a entrada de um companheiro atacante.

8) No futebol, um jogador está em posição de impedimento quando:

a) Se encontra na metade do campo adversário, mais próximo da linha de fundo e atrás da linha da bola.
b) Se encontra na metade do campo adversário, mais próximo da linha de fundo do que o penúltimo oponente e a bola.
c) Se encontra na metade do campo adversário, na mesma linha que o penúltimo oponente.

TESTE DE CONHECIMENTO TÁTICO OFENSIVO NO FUTEBOL

TCTOF-BRA

Parte 2

Assinale **uma resposta** em cada pergunta, aquela que você acredita que é a **mais adequada**. Considere as regras oficiais do futebol.

Sempre se pergunte **o que fazer se você é o jogador cinza**, ou seja, para responder **você deve imaginar que é o jogador cinza**.

Legenda: Atacante   Atacante cinza   ● Bola   ◄ Defensor   Meta/Gol

Da questão 9 a 14 você deve ler o enunciado, analisar a figura e responder **o que é mais adequado naquela situação de jogo**, ou seja, o jogador cinza (você) **naquele momento deve**: **a)** manter/conservar a posse de bola, **b)** avançar/progredir em direção ao gol adversário ou **c)** atacar a meta adversária/tentar marcar o gol? **Depois** de assinalar a melhor resposta, **você deve escolher "como" fazer isso**, por meio de uma das três opções apresentadas abaixo da frase "Realizando para isso:". **Sugestão:** antes de marcar **as respostas**, leia toda a questão.

EXEMPLO.

O que fazer se você é o jogador cinza que NÃO está com a bola?

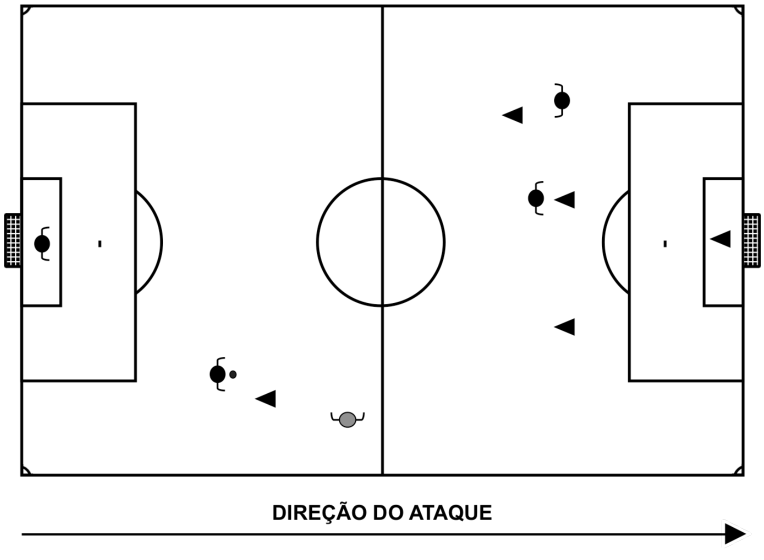

a) Ajudar a manter a posse de bola e não perdê-la.
✖ b) **Ajudar a avançar em direção ao gol adversário.** ⇦ Esta é a opção mais adequada.
c) Atacar a meta adversária.

Realizando para isso:

✖ a) **Um desmarque em direção ao gol adversário.** ⇦ Esta é a opção mais adequada.
b) Uma espera do passe, sem me movimentar.
c) Um desmarque em direção ao meu gol.

A segunda parte do teste começará na próxima página.

9. O que fazer se você é o jogador atacante com a bola?

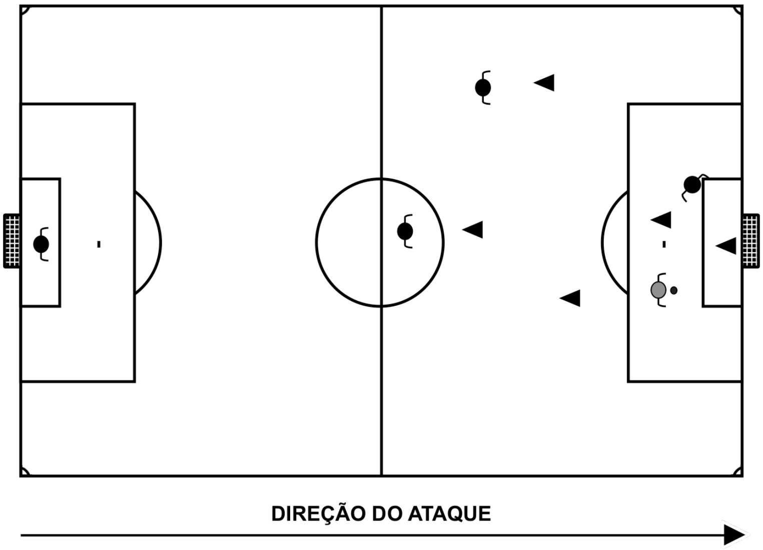

DIREÇÃO DO ATAQUE

a) Manter a posse de bola e não perdê-la.
b) Avançar em direção ao gol adversário.
c) Atacar a meta adversária.

Realizando para isso:

a) Um passe ao companheiro mais próximo.
b) Uma condução em direção ao gol adversário.
c) Um chute ao gol.

10. O que fazer se você é o jogador atacante que está com a bola?

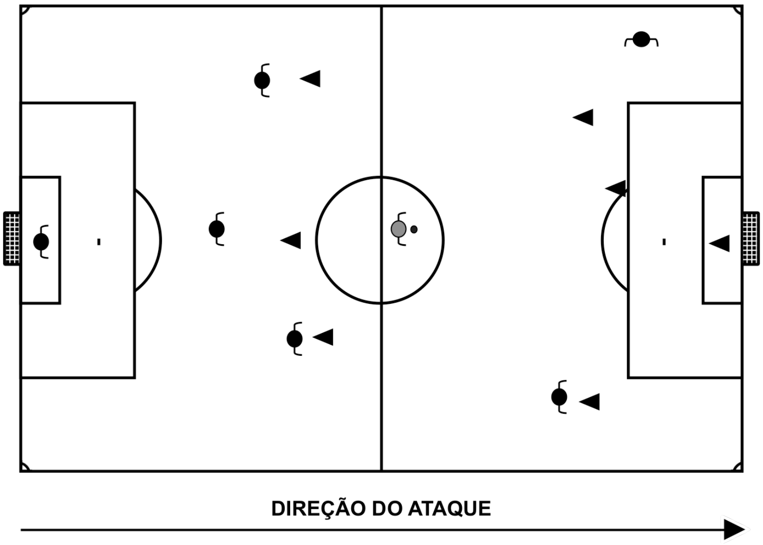

DIREÇÃO DO ATAQUE

a) Manter a posse de bola e não perdê-la.
b) Avançar em direção ao gol adversário.
c) Atacar a meta adversária.

Realizando para isso:

a) Um passe a um companheiro.
b) Uma condução em direção ao meu gol, até encontrar apoio de um companheiro.
c) Uma condução em direção ao gol adversário, até encontrar apoio de um companheiro ou ter a possibilidade de finalizar.

11. O que fazer se você é o jogador cinza que NÃO está com a bola?

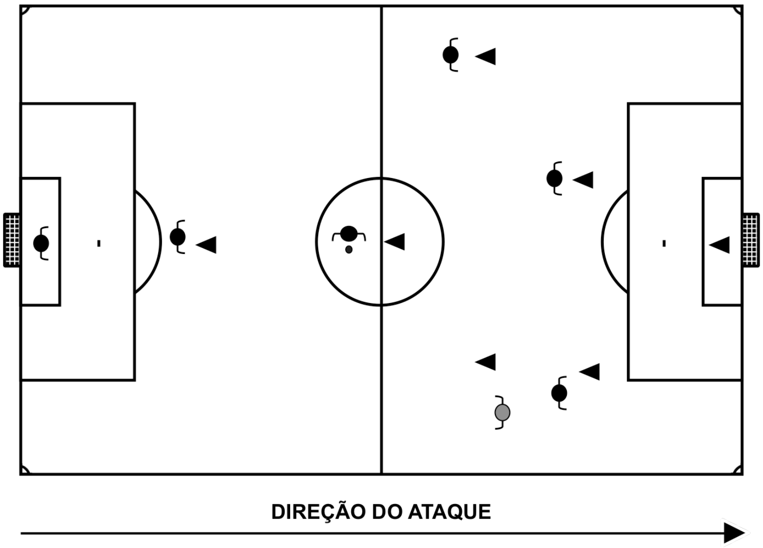

a) Ajudar a manter a posse de bola e não perdê-la.
b) Ajudar a avançar em direção ao gol adversário.
c) Ajudar a atacar a meta adversária.

Realizando para isso:

a) Um desmarque em direção ao gol adversário.
b) Uma espera do passe, sem me movimentar.
c) Um desmarque em direção ao meu gol.

12. O que fazer se você é o jogador atacante que está com a bola?

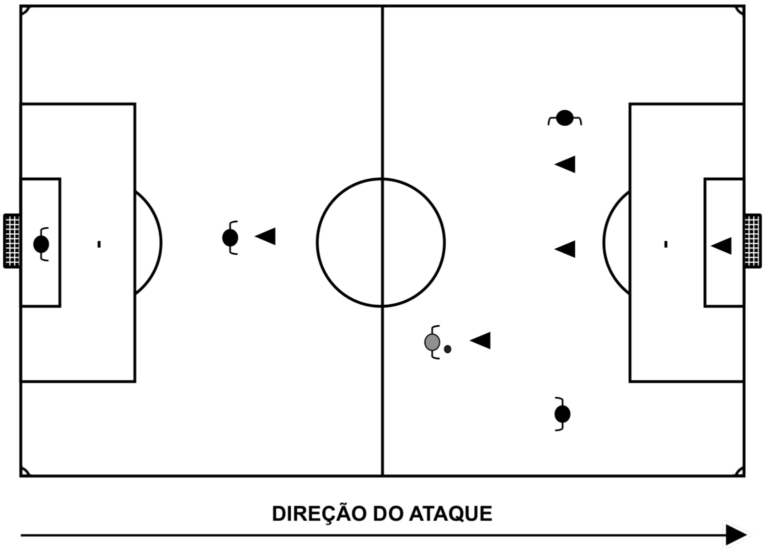

a) Manter a posse de bola e não perdê-la.
b) Avançar em direção ao gol adversário.
c) Atacar a meta adversária.

Realizando para isso:

a) Um passe ao companheiro que não está marcado por um defensor.
b) Uma condução ou drible até encontrar apoio de um companheiro.
c) Um chute ao gol.

13. O que fazer se você é o jogador atacante que está com a bola?

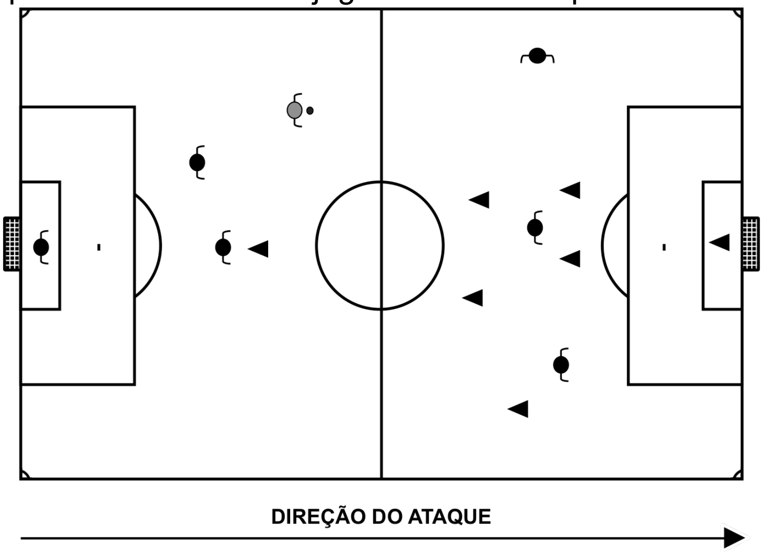

a) Manter a posse de bola e não perdê-la.
b) Avançar em direção ao gol adversário.
c) Atacar a meta adversária.

Realizando para isso:

a) Um passe ao companheiro adiantado que não está marcado por um defensor.
b) Uma condução até encontrar apoio de um companheiro.
c) Um chute ao gol.

14. O que fazer se você é o jogador atacante cinza que NÃO está com a bola?

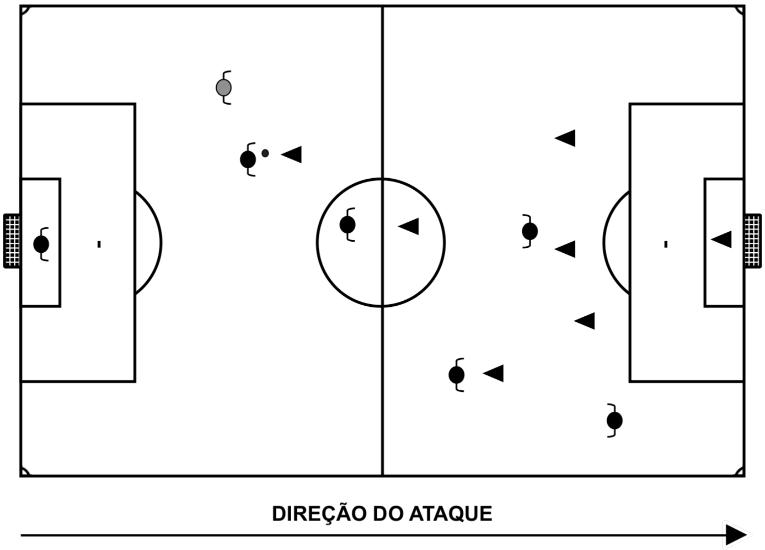

a) Ajudar a manter a posse de bola e não perdê-la.
b) Ajudar a avançar em direção ao gol adversário.
c) Ajudar a atacar a meta adversária.

Realizando para isso:

a) Um deslocamento em direção ao gol adversário.
b) Uma espera do passe, sem me movimentar.
c) Um deslocamento em direção ao meu gol.

15. Observe as quatro imagens correspondentes ao momento de um passe para o jogador cinza. Se você fosse esse jogador (cinza), em quais imagens **você estaria em posição de impedimento?** Pode ter **UMA ou MAIS** opções corretas. Assinale-a(s).

a)

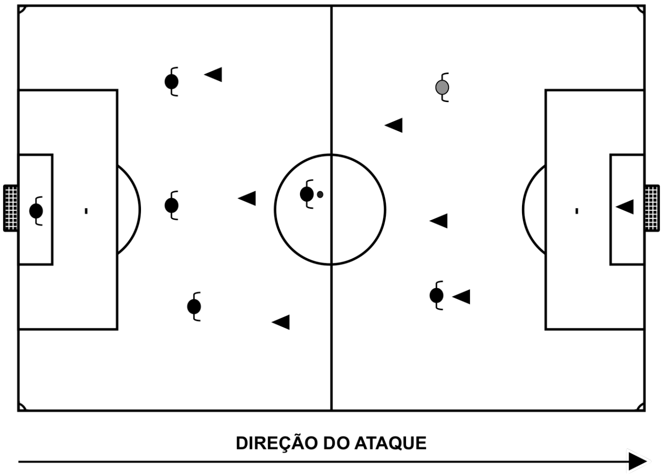

b)

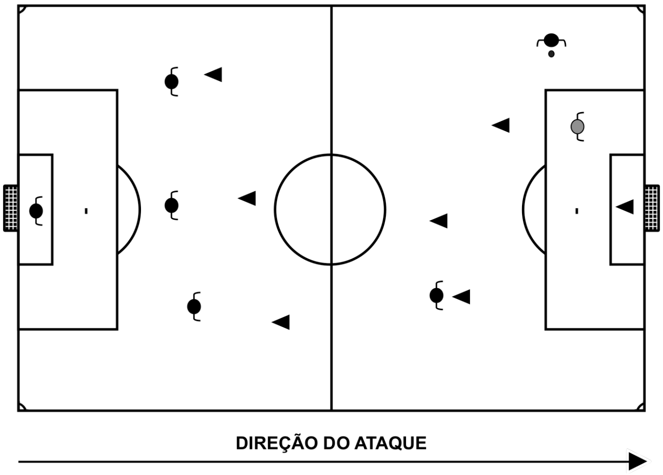

c)

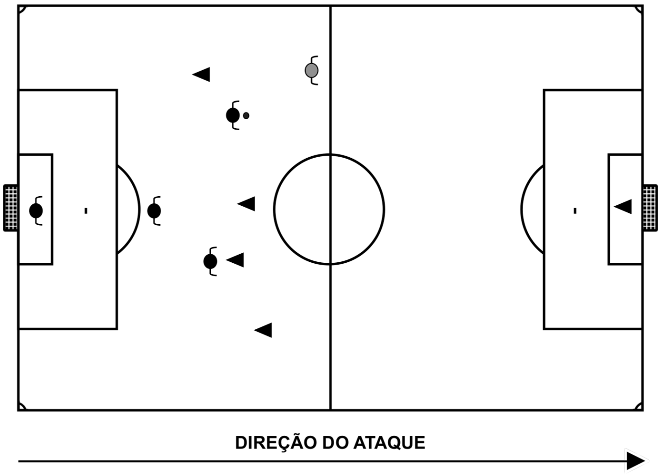

d)

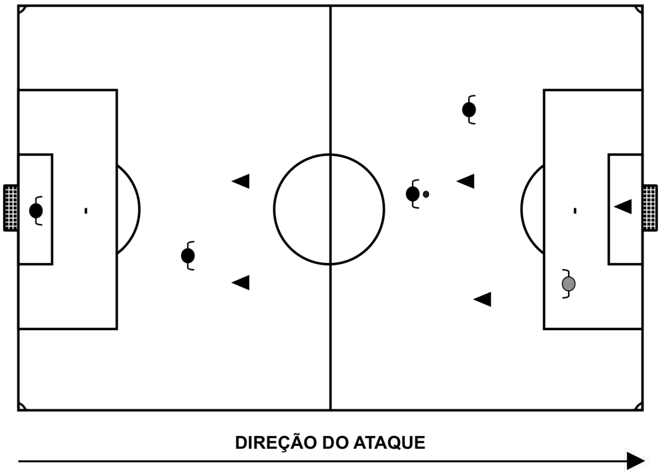

Atenção.
Confira se não deixou nenhuma questão em branco.

TESTE DE CONHECIMENTO TÁTICO OFENSIVO NO FUTEBOL

TCTOF-BRA

GABARITO

**Q1**	**Q2**	**Q3**	**Q4**	**Q5**	**Q6**	**Q7**	**Q8**
A	B	C	C	B	A	C	B
**Q9**	**Q10**	**Q11**	**Q12**	**Q13**	**Q14**	**Q15**	
CC	BC	AC	BA	BA	BA	BD	

Q = questão.

Nota: da Q9 a Q15 para acertar a questão o participante precisa responder corretamente as duas partes da questão. Exemplo: a questão 10 só estará correta se a resposta for “B” na primeira parte e “C” na segunda; em caso de qualquer outra combinação a resposta estará incorreta.

ORIENTAÇÕES

Cada resposta receberá **0** (zero) para resposta incorreta ou **1** (um) para resposta correta. Em cada uma das equações do TCTOF-BRA esse valor deverá ser multiplicado pela constante da questão. Por exemplo: se o participante errou Q1, Q2 e Q3 e acertou todas as outras, as primeiras três constantes serão multiplicadas por 0 (zero) e todas as outras constantes por 1 (um). Veja o exemplo abaixo para a equação “tactical knowledge”:

Tactical knowledge (pontos) = (0.045x**0** + 0.214x**0** + 0.301x**0** + 0.337x**1** + 0.083x**1** + 0.255x**1** + 0.07x**1** + 0.315x**1** + 0.403x**1** + 0.295x**1** + 0.125x**1** + 0.559x**1** + 0.341x**1** + 0.179x**1** + 0.252x**1**) x (10/3.774)

O mesmo procedimento deve ser adotado para os fatores 1, 2, 3 e 4, conforme equações apresentadas no artigo de validação.

Caso tenha interesse em receber uma planilha já preparada para a tabulação dos dados, faça uma solicitação por e-mail (lrechenchosky@uem.br ou rechenchosky@yahoo.com.br) com o título “Planilha de dados (TCTOF-BRA)”.
