# Supplementary material for: Adaptation and Validation of a Test for the Evaluation of Tactical Knowledge in Soccer: Test de Conocimiento Táctico Ofensivo en Fútbol for the Brazilian Context (TCTOF-BRA)
Source: Front Psychol. 2022 Jul 14;13:849255. doi: 10.3389/fpsyg.2022.849255 (PMC9330055; doi:10.3389/fpsyg.2022.849255)
Supplement: Supplementary file 3 [file Data_Sheet_3.pdf]

TEST OF TACTICAL OFFENSIVE KNOWLEDGE IN SOCCER: ENGLISH VERSION OF TCTOF-BRA[1]

I) Full name: ____

II) Club/institution: ____

III) Date of birth: ____/____/______ Date of evaluation: ____/____/______

IV) City and state of birth: ____

V) How long have you been training in soccer (don't consider futsal; only soccer)?

Less than 1 year ☐ 3 to 4 years ☐ 6 to 7 years ☐ 9 to 10 years ☐
1 to 2 years ☐ 4 to 5 years ☐ 7 to 8 years ☐ More than 10 years ☐
2 to 3 years ☐ 5 to 6 years ☐ 8 to 9 years ☐

I don't regularly participate in soccer training ☐ ← *If you marked this option, go to question IX)*

VI) Position that you normally play: ____

VII) How many times a week do you train in soccer? 1 ☐ 2 ☐ 3 ☐ 4 ☐ 5 ☐ 6 ☐ 7 ☐

VIII) What is the duration of each training session? 1h ☐ 1h15 ☐ 1h30 ☐ 2h ☐ Other:__

IX) Have you ever trained or do you train (regularly) in another sports modality?
No ☐ Yes ☐ If yes, which? ____

Part 1

Attention!

For questions 1 to 8 there is only ONE correct answer. Mark it with an "X" or "O".

1) What do you understand about keeping ball possession?

a) Maintaining ball possession and not losing it.
b) Progressing towards the opponent's goal.
c) Trying to score a goal.

2) What do you understand about moving towards the opponent's goal?

a) Maintaining ball possession and not losing it.
b) Progressing towards the opponent's goal.
c) Trying to score a goal.

[1] This is an English version of the TCTOF-BRA, requested by Frontiers in Psychology and translated by a committee composed of the 1st author, 2nd author - who is certified by the United States Soccer Federation D Coaching License, and a translator - who has English as a native language and proficiency in the Portuguese language.

3) What do you understand about attacking the opponent's goal?

a) Maintaining ball possession and not losing it.
b) Progressing towards the opponent's goal.
c) Trying to score a goal.

4) Providing “width" in the attack is?

a) Setting the ball in motion using an individual or collective strategy.
b) The players' movement in the attack towards the opponent's goal, using the play-space reasonably.
c) The players' movement in the attack, increasing the distance between them “to the touch lines” and using the play-space reasonably.

5) Providing “depth" in the attack is?

a) Setting the ball in motion using an individual or collective strategy.
b) The players' movement in the attack towards the opponent's goal, using the play-space reasonably.
c) The players' movement in the attack, increasing the distance between them “to the touch lines” and using the play-space reasonably.

6) Creating numeric superiority situations in attack are:

a) Players' movements in the attack, with the purpose of achieving a game situation where there are more attackers than defenders.
b) Players' movements in the attack, with and without the ball, creating free spaces.
c) Players' movements in the attack, with the purpose of achieving a game situation where there are more defenders than attackers.

7) Creating free spaces are:

a) Players' movements in the attack, with the purpose of achieving a game situation where there are more attackers than defenders.
b) Players' movements in the attack, decreasing the distance between them and using the play-space reasonably.
c) Movements in the attack, that provoke the defenders to move out of the occupying zones, in order to facilitate the entrance of a fellow attacker.

8) In soccer, a player is in offside position when they are:

a) In the opponent's half, nearer to the opponent's goal line and behind the ball.
b) In the opponent's half, nearer to the opponent's goal line than both the ball and the second-last opponent.
c) In the opponent's half, in the same line as the second-last opponent.

TEST OF TACTICAL OFFENSIVE KNOWLEDGE IN SOCCER:

ENGLISH VERSION OF TCTOF-BRA

Part 2

Mark **one answer** for each question, the one you believe to be the **most appropriate**. Consider the official rules of the soccer game.

Always ask yourself **what you would do if you were the gray player**, that is, to answer **you must imagine that you are the gray player.**

Labels: Attacker Gray attacker Ball Defender Goal

From question 9 to 14 you need to read the title, analyze the figure and answer **which option is the most appropriate in that game situation**, in other words, the gray player (you) **at that moment should: a)** keep/maintain the ball possession, **b)** advance/progress towards the opponent's goal, or **c)** attack the opponent's goal/try to score a goal? **After** marking the best answer, **you need to choose "how" to do this**, by choosing one of the three options shown below the phrase "Achieve this by:". **Suggestion:** before marking **the answers**, read the whole question.

EXAMPLE.

What do you do if you are the gray player who does NOT have the ball?

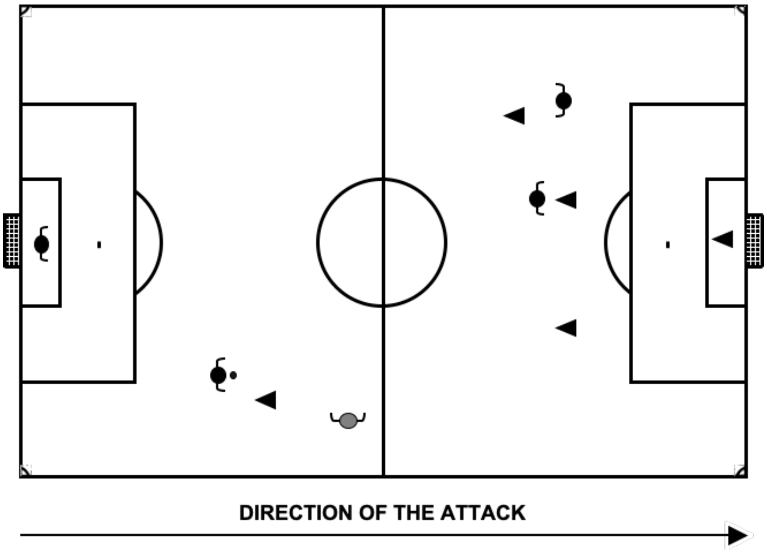

a) Help to maintain ball possession and don't lose it.
✖ b) Help to advance towards the opponent's goal. ⇦ This is the most appropriate answer.
c) Attack the opponent's goal.

Achieve this by:

✖ a) Unmarking towards the opponent's goal. ⇦ This is the most appropriate answer.
b) Waiting for a pass, without moving.
c) Unmarking towards my own goal.

The second part of the test starts on the next page.

9. What do you do if you are the attacking player with the ball?

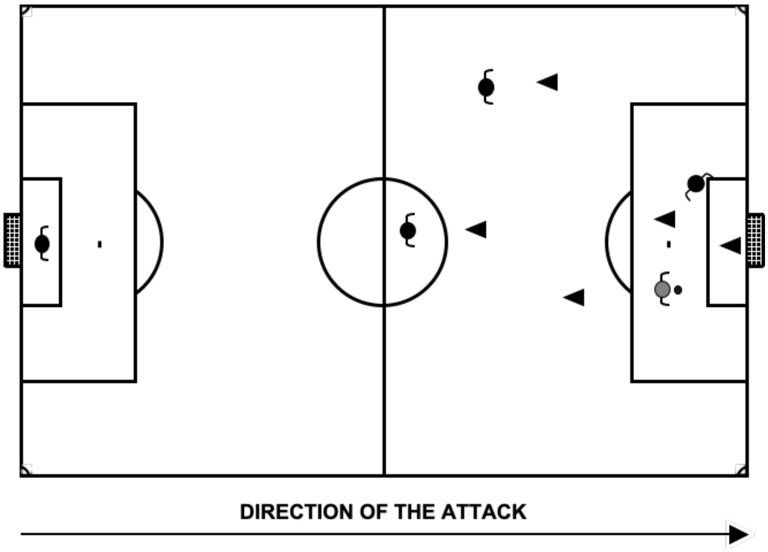

a) Maintain ball possession and don't lose it.
b) Advance towards the opponent's goal.
c) Attack the opponent's goal.

Achieve this by:

a) Passing to the closest teammate.
b) Carrying the ball towards the opponent's goal.
c) Shooting towards the goal.

10. What do you do if you are the attacking player with the ball?

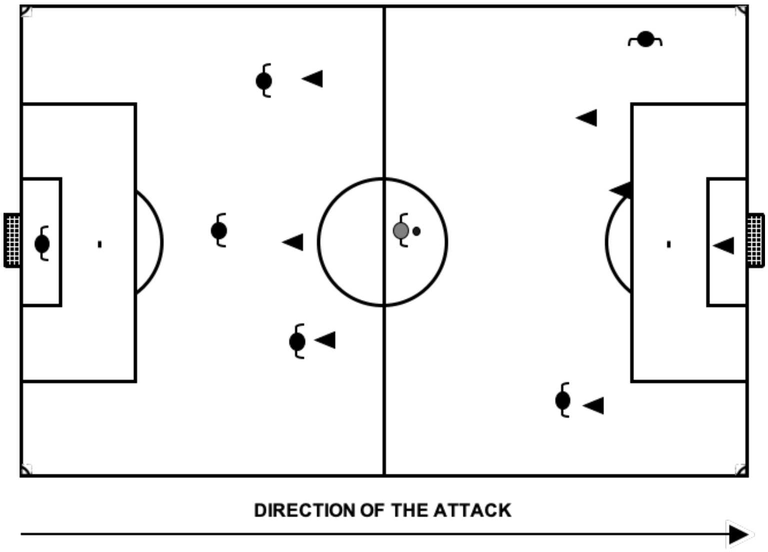

a) Maintain ball possession and don't lose it.
b) Advance towards the opponent's goal.
c) Attack the opponent's goal.

Achieve this by:

a) Passing to a teammate.
b) Carrying the ball towards my own goal until finding support from a teammate.
c) Carrying the ball towards the opponent's goal until finding support from a teammate or having the possibility to shoot.

11. What do you do if you are the gray player who does NOT have the ball?

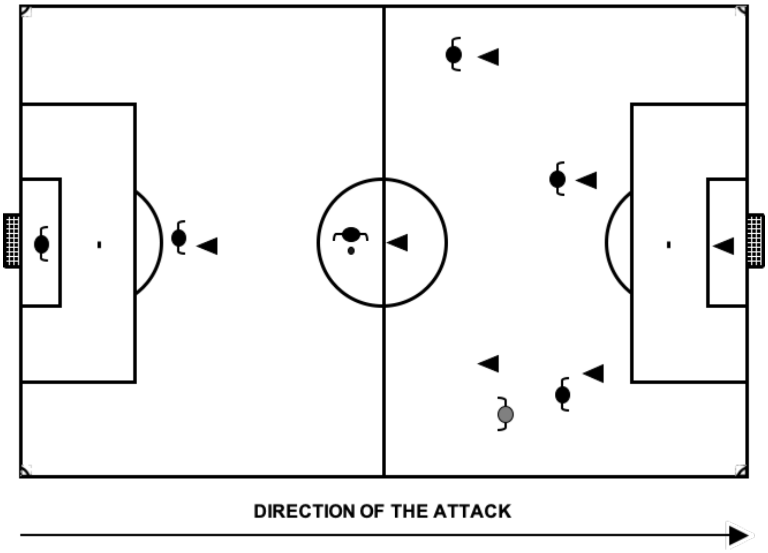

a) Help to maintain ball possession and don't lose it.
b) Help to advance towards the opponent's goal.
c) Help to attack the opponent's goal.

Achieve this by:

a) Unmarking towards the opponent's goal.
b) Waiting for a pass, without moving.
c) Unmarking towards my own goal.

12. What do you do if you are the attacking player with the ball?

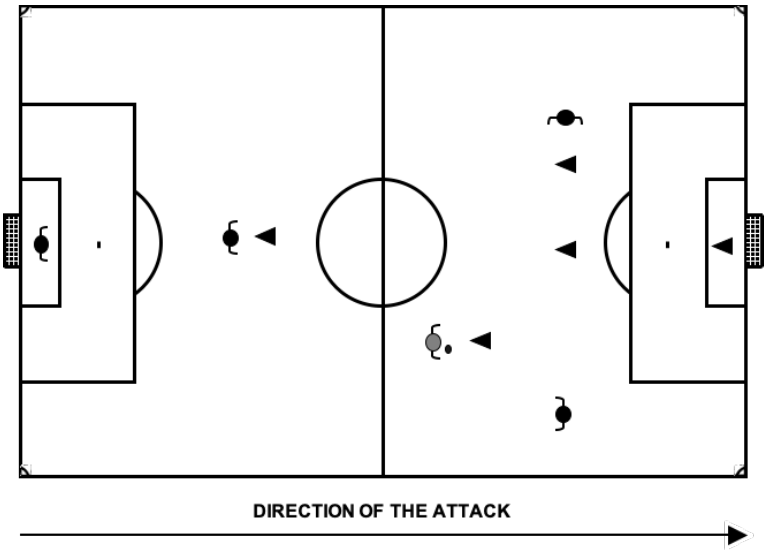

a) Maintain ball possession and don't lose it.
b) Advance towards the opponent's goal.
c) Attack the opponent's goal.

Achieve this by:

a) Passing to the teammate who is not being marked by a defender.
b) Carrying or dribbling the ball until finding support from a teammate.
c) Shooting towards the goal.

13. What do you do if you are the attacking player with the ball?

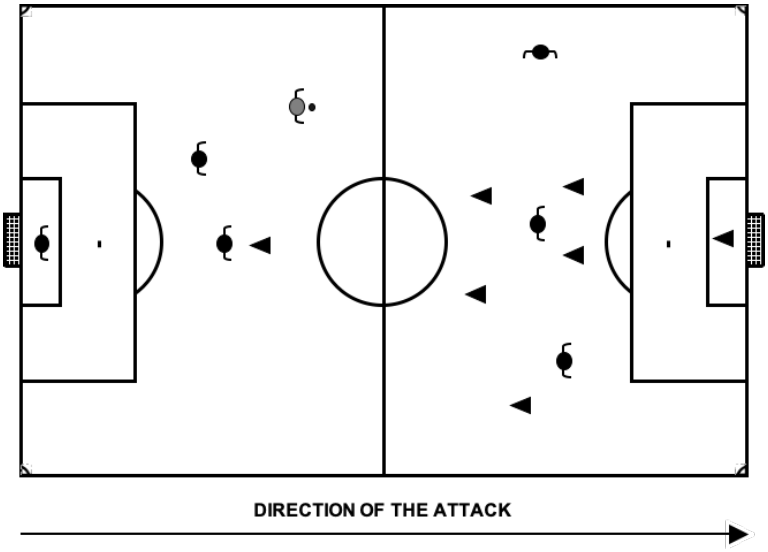

a) Maintain ball possession and don’t lose it.
b) Advance towards the opponent’s goal.
c) Attack the opponent’s goal.

Achieve this by:

a) Passing to the teammate ahead who is not being marked by a defender.
b) Carrying the ball until finding support from a teammate.
c) Shooting towards the goal.

14. What do you do if you are the gray attacking player who does NOT have the ball?

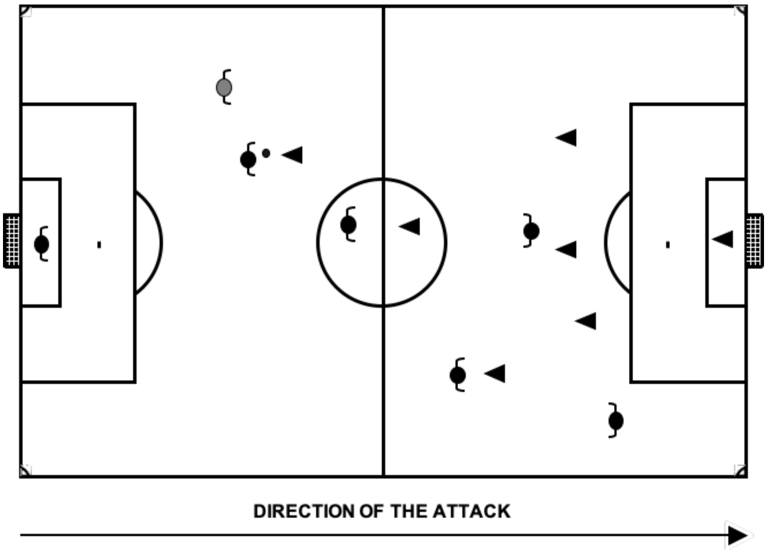

a) Help to maintain ball possession and don’t lose it.
b) Help to advance towards the opponent’s goal.
c) Help to attack the opponent’s goal.

Achieve this by:

a) Moving towards the opponent’s goal.
b) Waiting for a pass, without moving.
c) Moving towards my own goal.

15. Look at these four images corresponding to the moment of a pass to the gray player. If you were this player (gray), in which images **would you be in offside position? ONE or MORE** options may be correct. Mark it/them.

a)

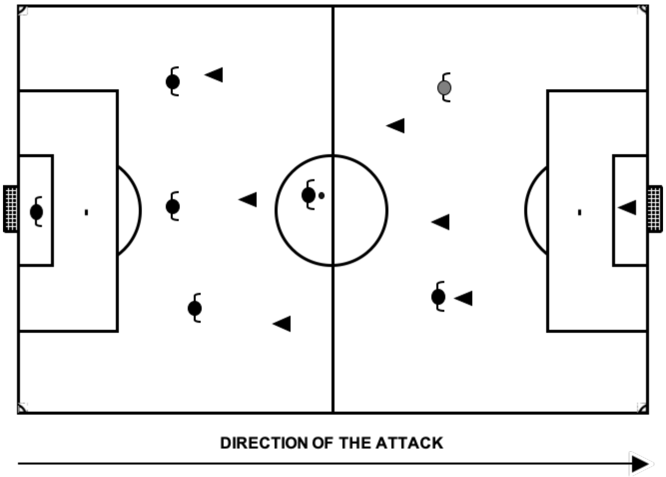

b)

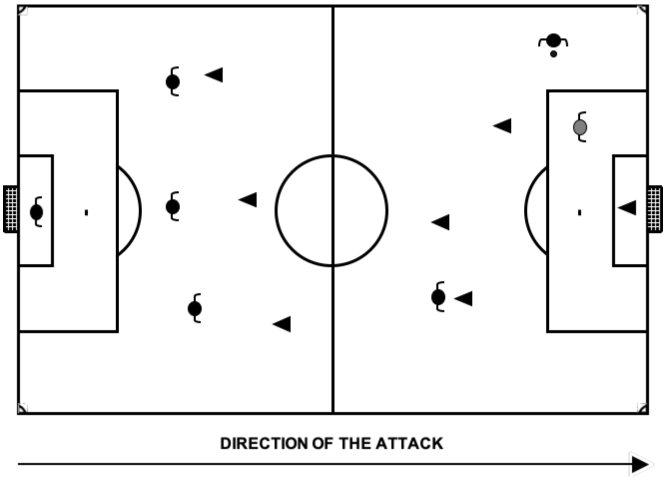

c)

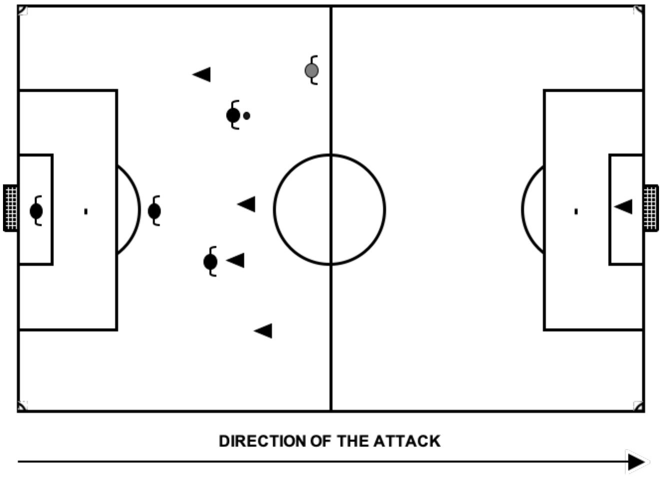

d)

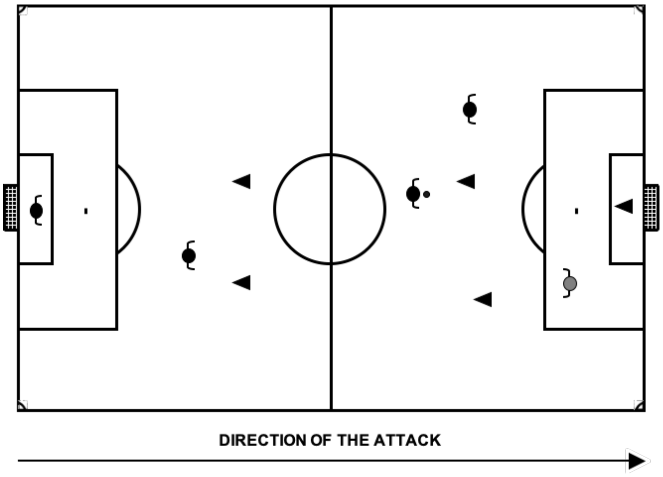

Warning.
Check that you didn't leave any question in blank.

TEST OF TACTICAL OFFENSIVE KNOWLEDGE IN SOCCER: ENGLISH VERSION OF TCTOF-BRA

ANSWER KEY

**Q1**	**Q2**	**Q3**	**Q4**	**Q5**	**Q6**	**Q7**	**Q8**
A	B	C	C	B	A	C	B
**Q9**	**Q10**	**Q11**	**Q12**	**Q13**	**Q14**	**Q15**	
CC	BC	AC	BA	BA	BA	BD	

Q = question.

Note: from Q9 to Q15 for the question to be correct the participant must choose the right answer in both parts of the question. Example: question 10 is only correct if the answer is "B" in the first part and "C" in the second; for any other combination, the answer will be incorrect.

GUIDELINES

Each answer receives 0 (zero) for an incorrect answer or 1 (one) for a correct answer. In each of the equations for TCTOF-BRA this value will have to be multiplied by the question constant. For example: if the participant answered Q1, Q2, and Q3 incorrectly and got all the other questions right, the first three constants will be multiplied by 0 (zero) and all the other constants by 1 (one). See the example below for the "tactical knowledge" equation:

Tactical knowledge (points) = (0.045x**0** + 0.214x**0** + 0.301x**0** + 0.337x**1** + 0.083x**1** + 0.255x**1** + 0.07x**1** + 0.315x**1** + 0.403x**1** + 0.295x**1** + 0.125x**1** + 0.559x**1** + 0.341x**1** + 0.179x**1** + 0.252x**1**) x (10/3.774)

The same procedure must be adopted for factors 1, 2, 3, and 4, according to the equations presented in the validation article.

In case you are interested in receiving a spreadsheet already prepared for data analysis, make a request by e-mail (lrechenchosky@uem.br or rechenchosky@yahoo.com.br) with the subject "Data Spreadsheet (TCTOF-BRA)".
